# Identification of reproductive and molecular biomarkers associated with clinical metritis in dromedary camels (*Camelus dromedarius*)

**DOI:** 10.3389/fvets.2026.1890020

**Published:** 2026-07-22

**Authors:** Haifa Ali Alqhtani, Ahmed A. Elsayed, Tahani M. I. Al-Hazani, Rasha yassin Elkhidr, Elshymaa A. Abdelnaby, Adel I. Almubarak, Mohammed Al-Rasheed, Ibrahim A. Emam, Fatmah Ahmed Safhi, Rowa K. Zarah, Ahmed Ateya, Mohamed Marzok

**Affiliations:** 1 Department of Biology, College of Science, Princess Nourah bint Abdulrahman University, Riyadh, Saudi Arabia; 2 Department of Animal and Poultry Nutrition, Desert Research Center, Cairo, Egypt; 3 Biology Department, College of Science and Humanities, Prince Sattam bin Abdulaziz University, Al-Kharj, Saudi Arabia; 4 Department of Clinical Studies, College of Veterinary Medicine, King Faisal University, Al-Ahsa, Saudi Arabia; 5 Department of Biology, College of Science, Taif University, Taif, Saudi Arabia; 6 Department of Development of Animal Wealth, Faculty of Veterinary Medicine, Mansoura University, Mansoura, Egypt

**Keywords:** antioxidant, dromedary camels, gene polymorphism, immunity, metritis

## Abstract

**Background:**

Metritis is a major cause of infertility, contributing to repeat breeding, early embryonic loss, fetal mortality, and abortion.

**Methods:**

This study investigated alterations in metabolic, hormonal, oxidative, immunological, and molecular markers associated with metritis susceptibility in dromedary camels (*Camelus dromedarius*). A total of 100 non-lactating multiparous females (87 healthy and 13 metritic) were examined. Blood samples were collected and analyzed to evaluate hematobiochemical parameters, inflammatory and antioxidant markers, and single nucleotide polymorphisms (SNPs) in selected immune- and antioxidant-related genes.

**Results:**

Metritic animals exhibited fever and abnormal uterine discharge. Gene expression analysis showed significant upregulation of *CARD9, SIGLEC1, VSIG4, BTRC, SLC2A3, ACOD1*, and *PFKFB3*, alongside downregulation of *CPT1A, ATF4, GADD45B, SLC7A11*, and *PRDX3*. Sequencing identified 19 SNPs, with significant differences in genotype distribution between groups, and discriminant analysis achieved complete classification accuracy. Hematological findings included normocytic normochromic anemia, leukocytosis, neutrophilia, lymphopenia, and monocytosis. Biochemically, metritic camels showed hypoglycemia and decreased levels of estrogen, progesterone, FSH, LH, T4, calcium, and antioxidant markers (GSH, CAT, and TAC), while NEFA, BHBA, cortisol, MDA, and pro-inflammatory cytokines (IL1α, IL1β, IL6, IL10, and TNFα) were significantly elevated.

**Conclusion:**

Overall, clinical metritis in dromedary camels was associated with significant hematological, metabolic, hormonal, oxidative, immunological, and genetic alterations. These findings identify a panel of reproductive and molecular biomarkers that may serve as promising candidate biomarkers associated with clinical metritis. However, given the observational nature of the study and the relatively small number of metritic she-camels, these findings should be interpreted with caution and require validation in larger, independent camel populations before their diagnostic, prognostic, or breeding applications can be established.

## Introduction

*Camelus dromedarius*, often known as the dromedary one-humped camel, is significant because it serves as a multifunctional animal that can be used for transport and work as well as to produce meat, milk, leather, and other byproducts (1, 2). However, seasonal pregnancy and birth rates in traditionally managed herds are generally lower than those observed in other domestic species (3). Because of their variable core temperatures and ability to survive for long periods without water, these animals thrive in the dry, semi-arid, and tropical climates found in Asia, Africa, and Oceania (4, 5).

Metritis is defined as inflammation involving all layers of the uterus, including the endometrium, submucosa, muscularis, and serosa (6). It is typically characterized by uterine enlargement accompanied by a fetid, brownish uterine discharge (7). Metritis is considered a major cause of infertility, contributing to repeat breeding syndrome, early embryonic loss, fetal death, and abortion (8, 9). Clinically affected animals often exhibit systemic signs such as fever, anorexia, weakness, reduced milk production, increased heart rate, and depression (6). The reported incidence of metritis in dromedary camels varies considerably depending on management practices and study design. Clinical puerperal metritis has been documented with a morbidity rate of approximately 13% in affected herds (10). Abattoir and field investigations have shown that uterine lesions, including metritis and endometritis, occur in about 13–30% of camels, with inflammatory conditions representing the most prevalent findings (11). Higher prevalence rates, reaching 45–66%, have been reported in infertile or repeat-breeder camels, highlighting the substantial burden of both clinical and subclinical uterine infections in this group (12). Routine examination of the genital tract based on history, external inspection, physical examination, palpation, ultrasonography, and vaginoscopy can provide valuable diagnostic information (13). However, these clinical methods are often limited by low sensitivity and specificity and may not accurately identify underlying fertility disorders (12). Therefore, uterine biopsy remains an important confirmatory diagnostic tool following initial clinical assessment (14).

Postpartum metritis is associated with multiple interrelated etiological factors that predispose animals to infection and generally require similar therapeutic approaches (15). During the peripartum period, animals undergo abrupt nutritional and endocrine changes that can impair immune function (16). This immunosuppression increases susceptibility to uterine infections (17). In this context, a reduced functional capacity of neutrophils has been documented in cows affected by metritis (18). Concurrently, alterations in metabolic status play a crucial role. Postpartum she-camels often experience negative energy balance (NEB), as feed intake fails to meet the high energy and protein demands required for milk production (19). Nutritional status is therefore a key determinant of immune competence in dairy animals (20). Indeed, an increased risk of metritis has been linked to NEB and associated metabolic disorders, such as ketosis and fatty liver, particularly during early lactation (21). Furthermore, the production and secretion of colostrum place a substantial demand on calcium reserves in the immediate postpartum period, potentially leading to hypocalcemia and reduced availability of ionized calcium (22). This decline in calcium levels has been associated with a higher likelihood of metritis diagnosis (23).

Previous research has indicated that plasma glucose concentrations during early lactation can be used as a reliable indicator for the diagnosis of metritis (24). Moreover, elevated serum non-esterified fatty acids (NEFA) during the week prior to parturition have been associated with an increased risk of metritis, retained placenta, and displaced abomasum after calving (25). Likewise, higher postpartum levels of NEFA and β-hydroxybutyrate (BHBA) have been linked to a greater incidence of metritis, as well as clinical ketosis and retained placenta (26).

Oxidative stress often occurs when there is an imbalance between antioxidants and oxidants, such as free radicals and reactive oxygen species. However, animal bodies can regulate excess free radicals through their defense mechanisms (27, 28). Catalases and other antioxidant enzymes are crucial for these processes (29). During uterine infection, activated neutrophils and macrophages produce large quantities of reactive oxygen species (ROS) as part of the innate immune response to eliminate the invading pathogens. However, excessive ROS production overwhelms the antioxidant defense system, leading to oxidative stress, lipid peroxidation, protein oxidation, DNA damage, and disruption of endometrial integrity, thereby contributing to the development and progression of metritis (28, 30, 31).

Oxidative stress associated with metritis has been demonstrated in several biological systems. In the blood, cows with metritis exhibit significantly increased concentrations of oxidative stress biomarkers, including malondialdehyde (MDA) and reactive oxygen metabolites, together with reduced total antioxidant capacity (TAC) and decreased activities of antioxidant enzymes, such as superoxide dismutase (SOD), catalase (CAT), and glutathione peroxidase (GPx) (30, 32). Similarly, circulating oxidative stress biomarkers are significantly elevated in dairy cows diagnosed with metritis, supporting their potential use as indicators of disease severity (33). Within the uterus, inflammatory exudates and uterine secretions contain increased concentrations of ROS, pro-inflammatory cytokines, chemokines, acute-phase proteins, and lipid peroxidation products, reflecting intense local inflammatory activity and oxidative damage that compromise endometrial repair and uterine function (30, 34). Furthermore, oxidative stress is not restricted to the reproductive tracts. Alterations in milk oxidative biomarkers, including increased lipid oxidation and reduced antioxidant capacity, have been reported in postpartum dairy cows suffering from inflammatory disorders, indicating that systemic oxidative imbalance can also impair mammary gland function and milk quality (32).

Marker-assisted selection (MAS) has emerged as a valuable genomic approach for improving disease resistance by identifying favorable alleles associated with health traits, thereby increasing selection accuracy, independently of environmental influences (35–38). However, the successful application of MAS depends on the identification of reliable molecular markers associated with resistance to disease.

Given the multifactorial nature of metritis, genes involved in innate immunity, inflammatory signaling, immunometabolism, oxidative stress regulation, and cellular stress responses may contribute to its susceptibility and progression. Therefore, *CARD9, SIGLEC1, VSIG4*, and *BTRC* were selected based on their established roles in pathogen recognition, macrophage activation, and inflammatory signaling. *ACOD1, SLC2A3, CPT1A*, and *PFKFB3* are markers of immunometabolic reprogramming and energy metabolism (30, 39, 40). *ATF4, GADD45B,* and *SLC7A11* were investigated because of their involvement in cellular stress adaptation and redox homeostasis, whereas *PRDX3* was selected as a key mitochondrial antioxidant defense gene (39, 41). Collectively, these genes represent biologically relevant pathways potentially associated with metritis susceptibility in pregnant dromedary camels.

This study aimed to investigate the clinical, hematobiochemical, hormonal, oxidative stress, inflammatory, transcriptional, and genetic biomarkers associated with metritis susceptibility in dromedary she-camels. Particular emphasis was placed on evaluating the expression profiles and genetic polymorphisms of selected immune, metabolic, stress response, and antioxidant-related genes and their relationships with disease status.

## Materials and methods

### Animals, study area, and experimental design

This study included 100 multiparous she-camels (*Camelus dromedarius*) reared under similar management conditions in Siwa Oasis, Egypt. At the beginning of the study, all animals were non-lactating and had recently calved. The camels had a mean body weight of 512 kg (range: 390–634 kg) and mean age of 20 years (range: 18–22 years). Before enrollment, all animals underwent a complete clinical examination to assess their general health. Camels with evidence of systemic illness, metabolic disorders, reproductive abnormalities unrelated to postpartum uterine disease, or other concurrent infectious diseases were excluded. In addition, camels with dystocia requiring extensive obstetrical intervention, retained fetal membranes persisting beyond the normal postpartum period, traumatic injuries of the reproductive tract, or those that had received antimicrobial or anti-inflammatory treatment before sample collection were excluded to minimize the potential confounding effects on the evaluated biomarkers.

The experiment was conducted in Siwa Oasis, Egypt (29°06′–29°24′N, 25°16′–26°12′E), approximately 330 km southwest of the Mediterranean Coast and 65 km east of the Libyan border. The region is characterized by a typical hyper-arid desert climate with high ambient temperatures, low annual rainfall, low relative humidity, and significant daily temperature fluctuations. Camel production in this region relies primarily on extensive grazing of natural desert vegetation, supplemented with cultivated forages and concentrated feeds according to seasonal forage availability.

### Animal management

Throughout the study, all camels were maintained under open-yard housing conditions and managed in a semi-intensive production system. Animals grazed on natural rangelands during the day and received supplementary feeding to satisfy their nutritional requirements. Each camel was provided with 3 kg/day of a commercial concentrate mixture formulated to supply approximately 50% of the maintenance energy requirements. The concentrate consisted of 55% ground corn, 15% soybean meal, 10% cottonseed meal, 15% wheat bran, 2.5% limestone, 1.5% sodium chloride, 0.5% sodium bicarbonate, 0.1% yeast culture, 0.1% antitoxin preparation, and 0.3% vitamin–mineral premix, as previously described (42). Egyptian clover hay (*Trifolium alexandrinum*) and fresh drinking water were provided ad libitum throughout the experimental period.

Routine preventive healthcare was equally applied to all animals. Camels were periodically dewormed using albendazole administered orally at a dose of 3.8–15 mg/kg body weight as a broad-spectrum anthelmintic. In addition, prophylactic treatment against trypanosomiasis was administered twice annually using a combination of quinapyramine sulfate and quinapyramine chloride (Triquin, Wockhardt, Mumbai, and India).

### Clinical monitoring and diagnosis of postpartum metritis

Following parturition, all she-camels were monitored daily for 21 days postpartum for the development of clinical metritis (CM). Clinical examinations were performed by the same experienced veterinarian throughout the study to minimize variations in observations. Each examination included assessment of rectal temperature, heart rate, respiratory rate, appetite, general health status, and rectal palpation of the reproductive tract to evaluate uterine involution, uterine wall consistency, and the presence of inflammation.

Postpartum metritis was diagnosed based on clinical findings, including fever, increased heart and respiratory rates, delayed uterine involution with enlarged and tense uterine walls, and the presence of abnormal fetid uterine discharge. The discharge was characterized according to its color (milky gray, brown, or blood-tinged) and consistency (muco-purulent, purulent, or watery). Animals that exhibited these clinical signs within the first 21 days postpartum were classified as having metritis. All biological samples were collected immediately after the diagnosis of clinical metritis and before initiating any therapeutic intervention. Bacteriological culture was not performed because the primary objective of the present study was to characterize host hematological, biochemical, hormonal, oxidative stress, inflammatory, and molecular biomarkers associated with naturally occurring clinical metritis, rather than to identify etiological bacterial pathogens.

Among the 100 examined she-camels, 87 remained clinically healthy throughout the postpartum period and showed normal uterine involution, rectal temperature, appetite, and absence of abnormal uterine discharge. The remaining 13 camels fulfilled the diagnostic criteria for clinical metritis and were included in the metritis group for subsequent hematological, biochemical, oxidative stress, hormonal, and molecular analyses.

### Blood sampling

Approximately 10 mL of blood was aseptically collected from the jugular vein of each camel using sterile, disposable needles and vacutainer tubes. Immediately after collection, blood was divided into two portions. Five milliliters of blood was transferred into tubes containing ethylenediaminetetraacetic acid (EDTA) as an anticoagulant for hematological analysis and whole-blood RNA extraction, while the remaining 5 mL was placed into plain tubes without anticoagulant for serum separation. All samples were immediately placed in an ice box containing crushed ice and transported to the laboratory for processing. EDTA-anticoagulated whole blood was used for complete blood count (CBC) determination and total RNA extraction for subsequent gene expression analysis. Blood collected in plain tubes was allowed to clot overnight at room temperature and then centrifuged at 3000 rpm for 15 min to separate the serum. The separated serum was carefully aspirated, aliquoted into sterile microcentrifuge tubes to prevent repeated freeze–thaw cycles, and stored at −20 °C until biochemical analyses, including metabolic, oxidative stress, antioxidant, inflammatory, and hormonal biomarkers, were performed according to the respective assay protocols.

#### RNA extraction and quantitative real-time RT-PCR

Total RNA was isolated from whole blood samples using TRIzol reagent, followed by purification using the RNeasy Mini Kit (Qiagen, Hilden, Germany) in accordance with the manufacturer’s instructions (43). RNA concentration and purity were evaluated spectrophotometrically using a NanoDrop system, and RNA integrity was verified prior to downstream application.

Complementary DNA (cDNA) was synthesized from purified RNA using a reverse transcription kit (Thermo Fisher Scientific, USA). Quantitative real-time PCR (qRT-PCR) was performed using SYBR Green chemistry (SensiFAST SYBR Master Mix, Bioline, UK) under standardized conditions, following the Minimum Information for Publication of Quantitative Real-Time PCR Experiments (MIQE) guidelines (44).

Gene expression analysis targeted key genes involved in immune responses: caspase recruitment domain family member 9 (*CARD9*), sialic acid binding ig like lectin 1 (*SIGLEC1*), and v-set and immunoglobulin domain containing 4 (*VSIG4*); ubiquitination and signaling beta-transducin repeat containing E3 ubiquitin protein ligase (*BTRC*); metabolic pathways solute carrier family 2 member 3 (*SLC2A3*), aconitate decarboxylase 1 (*ACOD1*), carnitine palmitoyltransferase 1a (*CPT1A*), and 6-phosphofructo-2-kinase/fructose-2,6-bisphosphatase 3 (*PFKFB3*); stress response and redox regulation—activating transcription factor 4 (*ATF4*), growth arrest and dna damage inducible beta (*GADD45B*), and solute carrier family 7 member 11 (*SLC7A11*); and oxidative stress defense peroxiredoxin 3 (*PRDX3*). Gene-specific primers were designed based on the reference sequences of *Camelus dromedarius* retrieved from the GenBank database using Primer-BLAST tools (45). The primer sequences and amplification conditions are presented in Table 1.

**Table 1:** Forward and reverse oligonucleotide-based real-time PCR primers for the being investigated.

Investigated marker	Primer	Product size (bp)	Annealing temperature (°C)	GenBank isolate
*CARD9*	F5′-TACCTGCGGCAGTGCAAGGTC-3′ R5′-GCCTCGCAAGCCTCCTTGAGCC3′	413	55	XM_031450839.2
*SIGLEC1*	F5′-CTGCGCTGCCAGCTCTCGGTG-3 R5′-CTCAGCCATGAAGATGTGGAGG-3′	339	55	XM_010988253.3
*VSIG4*	F5′-CACTACACGTGTGAAGTCACTT −3′ R5′-CTCATCAGAGTAATTGTTGCCCA-3′	468	58	XM_010981998.3
*BTRC*	F5′-ATGGACCCGGCCGAGGCGGTAC-3′ R5′-AGATGTCACTCGGTACCATTCCT-3′	474	55	XM_031461630.2
*SLC2A3*	F5′- AGGTGGCGCAATTCAATGCTAA-3′ R5′-TCTGTTAATGAGCAAGAATCTC-3′	384	55	XM_031444155.2
*ACOD1*	F5′-ACGCTGCTTGATGGTGCCATC-3′ R5′- GAAGCCACTTCTGGTGGAGAGAG-3′	383	58	XM_010996663.3
*CPT1A*	F5′-ACAATTCCGCTCTGCTCTGCCCA-3′ R5′-TCCACGTCTTCCTTCCTGTATC-3′	401	58	XM_031448592.2
*PFKFB3*	F5′-GTCCGAAAGCAGTGTGCACTAG −3′ R5′-CTGCACGTGGATGTTCATTAGGT-3′	444	55	XM_064479318.1
*ATF4*	F5′-CTGAGCAGCGAGGTGTTGGTGG-3′ R5′-TCTGGGAGATGGCCAATTGGGT-3′	419	55	XM_010983554.3
*GADD45B*	F5′-GTGGCCGCGCAGCGCCAGGACC -3′ R5′-GACCCACTGGTTGTTCTCCCGGC-3′	375	58	XM_010997221.3
*SLC7A11*	F5′-GTCAGAAAGCCCGTTGTGTCCAC -3′ R5′-TGTAGCGTCCAAATGCCAGAG-3′	445	55	XM_031465945.2
*PRDX3*	F5′-CCTTATGCCTCTCGAAGAATGT −3′ R5′-TAGGGCCAGACCAGCACCTTCCA-3′	459	58	XM_010997880.3
*GAPDH*	F5′-GGCGTGAACCACGAGAAGTA-3′ R5′-GGCGTGGACTGTGGTCATAA −3′	141	58	XM_010990867.3

Caspase recruitment domain family member 9 (CARD9), sialic acid binding ig like lectin 1 (SIGLEC1), v-set and immunoglobulin domain containing 4 (VSIG4); beta-transducin repeat containing E3 ubiquitin protein ligase (BTRC); solute carrier family 2 member 3 (SLC2A3), aconitate decarboxylase 1 (ACOD1), carnitine palmitoyltransferase 1a (CPT1A), 6-phosphofructo-2-kinase/fructose-2,6-bisphosphatase 3 (PFKFB3); activating transcription factor 4 (ATF4), growth arrest DNA damage inducible beta (GADD45B), solute carrier family 7 member 11 (SLC7A11); and peroxiredoxin 3 (PRDX3).

Primer specificity was verified by the presence of a single amplification product of the expected size and a single peak in the melting curve analysis. Amplification efficiencies were assessed and ranged from 90 to 110% (44). *GAPDH* was selected as the reference gene because its Ct values exhibited minimal variation among samples and experimental groups, supporting stable expression under the study conditions, consistent with recommendations for the use of stably expressed housekeeping genes in qPCR normalization (46).

Amplification reactions were conducted in a thermal cycler under the following conditions: initial reverse transcription at 50 °C for 30 min, initial denaturation at 95 °C for 5 min, followed by 40 amplification cycles consisting of denaturation at 95 °C for 15 s, annealing at optimized temperatures for each primer set, and extension at 72 °C for 30 s. Melting curve analysis was performed at the end of each run to support the specificity of amplification. Relative gene expression levels were normalized to the housekeeping gene *GAPDH* and calculated using the comparative 2^−ΔΔCt method (47).

#### DNA sequencing and SNP detection

PCR amplicons exhibiting specific single bands were purified using a commercial PCR purification kit (Jena Bioscience, Germany) to eliminate residual primers and non-specific products (48). The concentration and quality of purified DNA were assessed using spectrophotometric analysis prior to sequencing.

Bidirectional DNA sequencing was performed using an ABI 3730XL automated sequencer (Applied Biosystems, USA) based on the Sanger sequencing method (49). The sequence quality of the raw sequencing chromatograms produced by bidirectional Sanger sequencing was first assessed using Chromas software (Technelysium Pty Ltd., Australia). Uncertain nucleotide calls were examined and adjusted depending on the chromatogram peak quality, and low-quality areas at both the 5′ and 3′ ends were manually cut. To increase the sequence accuracy, the forward and reverse sequences of each sample were combined into consensus sequences. Only nucleotide substitutions that exhibited clear and reproducible peaks in the bidirectional sequencing reads were considered true polymorphisms.

To verify gene identity and evaluate sequence homology with published *Camelus dromedarius* reference sequences, the obtained consensus sequences were subjected to similarity searches using the Basic Local Alignment Search Tool (BLASTn), which is accessible through the NCBI database. The ClustalW algorithm included in MEGA software (Version 11.0) was used for sequence alignment analyses. By comparing sequences from metritis and healthy she-camels to the appropriate reference sequences, single nucleotide polymorphisms (SNPs) were identified. True polymorphic sites were defined as nucleotide substitutions found in both forward and reverse sequencing reads, and were verified by several sequence alignments. SNP locations were noted in accordance with the GenBank reference sequences that corresponded to them. To identify the genetic differences between the groups under study, nucleotide variation patterns and sequence identity percentages were analyzed (50).

### Biochemical and hormonal analysis

Serum biochemical, metabolic, hormonal, and inflammatory biomarkers were analyzed according to the manufacturer’s instructions using commercially available diagnostic kits. Prior to analysis, the frozen serum samples were thawed once at room temperature, gently mixed, and analyzed immediately. All measurements were performed in duplicate, and the mean values were used for statistical analysis. Quality control sera supplied by the manufacturers were included in each analytical run to ensure the reliability of the assay.

Metabolic indicators of negative energy balance were evaluated by measuring serum beta-hydroxybutyrate (BHBA) using a colorimetric assay kit (Cayman Chemical, USA; Item No. 700190) and non-esterified fatty acids (NEFA) using a commercial kit (Randox Laboratories Ltd., Crumlin, Co. Antrim, UK).

Serum glucose and calcium concentrations were determined using enzymatic colorimetric methods with commercial diagnostic kits (Biodiagnostic Company, Egypt), and absorbance was measured with a UV–visible spectrophotometer according to the manufacturer’s recommended wavelengths and procedures.

Hormonal analyses were performed using enzyme-linked immunosorbent assay (ELISA) kits. Serum cortisol and estradiol concentrations were measured using Parameter ELISA kits (USA; Ref. KGE008B and KGE014, respectively). Progesterone levels were determined using an ELISA kit from Oxford Biomedical Research (USA; Ref. EA74). Serum thyroxine (T4) levels were quantified using a GenWay Biotech ELISA kit (USA; Catalog No. GWB-8C0053). Follicle-stimulating hormone (FSH) concentrations were measured using ELISA kits supplied by Enzo Life Sciences Inc. (USA), whereas luteinizing hormone (LH) levels were determined using a commercial ELISA kit from ELK Biotechnology (China).

The inflammatory profile was evaluated by measuring the serum concentrations of the pro-inflammatory cytokines interleukin-1 alpha (IL-1*α*), interleukin-1 beta (IL-1β), interleukin-6 (IL-6), and tumor necrosis factor-alpha (TNF-α), together with the anti-inflammatory cytokine interleukin-10 (IL-10), using species-validated commercial ELISA kits (MyBioSource, USA). Cytokine concentrations were calculated from standard calibration curves generated for each assay, according to the manufacturer’s instructions.

Oxidative stress and metabolic biomarkers, including malondialdehyde (MDA), catalase (CAT), reduced glutathione (GSH), total antioxidant capacity (TAC), glucose, and calcium, were measured spectrophotometrically using commercial diagnostic kits (Biodiagnostic Co., Giza, Egypt) according to the manufacturer’s instructions. The MDA concentration was determined based on the formation of thiobarbituric acid-reactive substances (TBARS), producing a pink chromogen measured at 534 nm. Catalase (CAT) activity was assessed by measuring the rate of decomposition of hydrogen peroxide (H₂O₂), with a decrease in absorbance recorded at 510 nm. The reduced glutathione (GSH) concentration was determined using 5,5′-dithiobis-(2-nitrobenzoic acid) (DTNB), which reacts with sulfhydryl groups to form a yellow-colored product measured at 405 nm. Total antioxidant capacity (TAC) was evaluated by measuring the ability of serum antioxidants to neutralize a defined amount of exogenously added hydrogen peroxide. The residual H₂O₂ reacts with a chromogenic substrate, and the resulting color intensity, measured at 505 nm, is inversely proportional to the antioxidant capacity of the sample.

For all ELISA assays, optical density was measured using a microplate reader at the recommended wavelength, and analyte concentrations were calculated from the standard curves using the accompanying software. All assays were conducted following the manufacturer’s standard operating procedures, and intra- and inter-assay quality controls were included to ensure analytical precision and reproducibility.

### Statistical analysis

Statistical analyses were performed using IBM SPSS Statistics version 23.0 (IBM Corp., Armonk, NY, USA). The distribution of each continuous variable was assessed using the Kolmogorov–Smirnov test and visual inspection of the Q–Q plots. Variables with *p* > 0.05 were considered normally distributed and are presented as mean ± standard error (SE).

Comparisons between healthy (*n* = 87) and metritic (*n* = 13) camels were performed using an independent-samples t-test. Because of the unequal sample sizes between groups, Welch’s t-test was conducted to account for the potential heterogeneity of variances. The results obtained from Welch’s *t*-test were consistent with those of the independent-samples t-test, confirming the robustness of the comparisons. To evaluate whether the available sample size provided sufficient statistical sensitivity, a *post hoc* power analysis was performed based on the primary outcome measures using the observed effect sizes with a significance level (*α*) of 0.05. The analysis indicated that the study achieved adequate statistical power to detect the differences between healthy and metritic camels.

To control for type I error resulting from multiple comparisons, *p*-values obtained from gene expression and biomarker analyses were adjusted using the Benjamini–Hochberg false discovery rate (FDR) procedure, with adjusted p-values < 0.05 considered statistically significant.

Differences in genotype and allele frequencies between healthy and metritic camels were evaluated using the chi-square test. The observed genotype frequencies were analyzed using the chi-square (χ^2^) test to evaluate compliance with the Hardy–Weinberg equilibrium (HWE) and to determine the distribution of genotypes within the study population (51, 52). Linear Discriminant Analysis (LDA) was performed to determine whether the average SNP scores of the 12 candidate genes could discriminate between healthy and metritic camels. The average SNP scores were used as predictor variables, and health status served as the grouping variable. To evaluate the stability of the discriminant model and minimize overfitting due to the relatively small sample size, leave-one-out cross-validation (LOOCV) was performed, and the cross-validated classification accuracy was reported.

The relationships between the relative expression levels of the investigated genes and the serum concentrations of hormonal, oxidative stress, antioxidant, and inflammatory biomarkers were evaluated using Pearson’s correlation analysis. Correlation coefficients (r) and *p*-values were reported. Statistical significance was defined as *p* < 0.05 after adjustment for multiple testing, where appropriate.

## Results

### Clinical findings

There was a significant (*p* < 0.05) increase in body temperature, pulse, and respiratory rates (40.5 ± 0.2 °C, 66 ± 0.5 beats/min, and 32±0.5 breaths/min), respectively, in metritic group in relation to control group (38.1 ± 0.2 °C, 48±0.5 beats/min, and 20±1.1 breaths/min), respectively (Table 2). The metritic group exhibited uterine inflammation, characterized by enlarged and tense uterine walls and abnormal discharge varying in color (milky gray, brown, or bloody) and consistency (muco-purulent, purulent, or watery).

**Table 2:** Mean values (M±SE) of temperature, pulse and respiration in control (*n* = 87) and diseased group (*n* = 13) she-camels.

Variables	Control animals	Diseased animals	P-value
Temperature (°C)	38.1 ± 0.2	40.5 ± 0.2*	0.001
Pulse (beats/min)	48±0.5	66 ± 0.5*	0.001
Respiration (breaths/min)	20±1.1	32±0.5*	0.001

### Hematological findings

**Figure 1:**
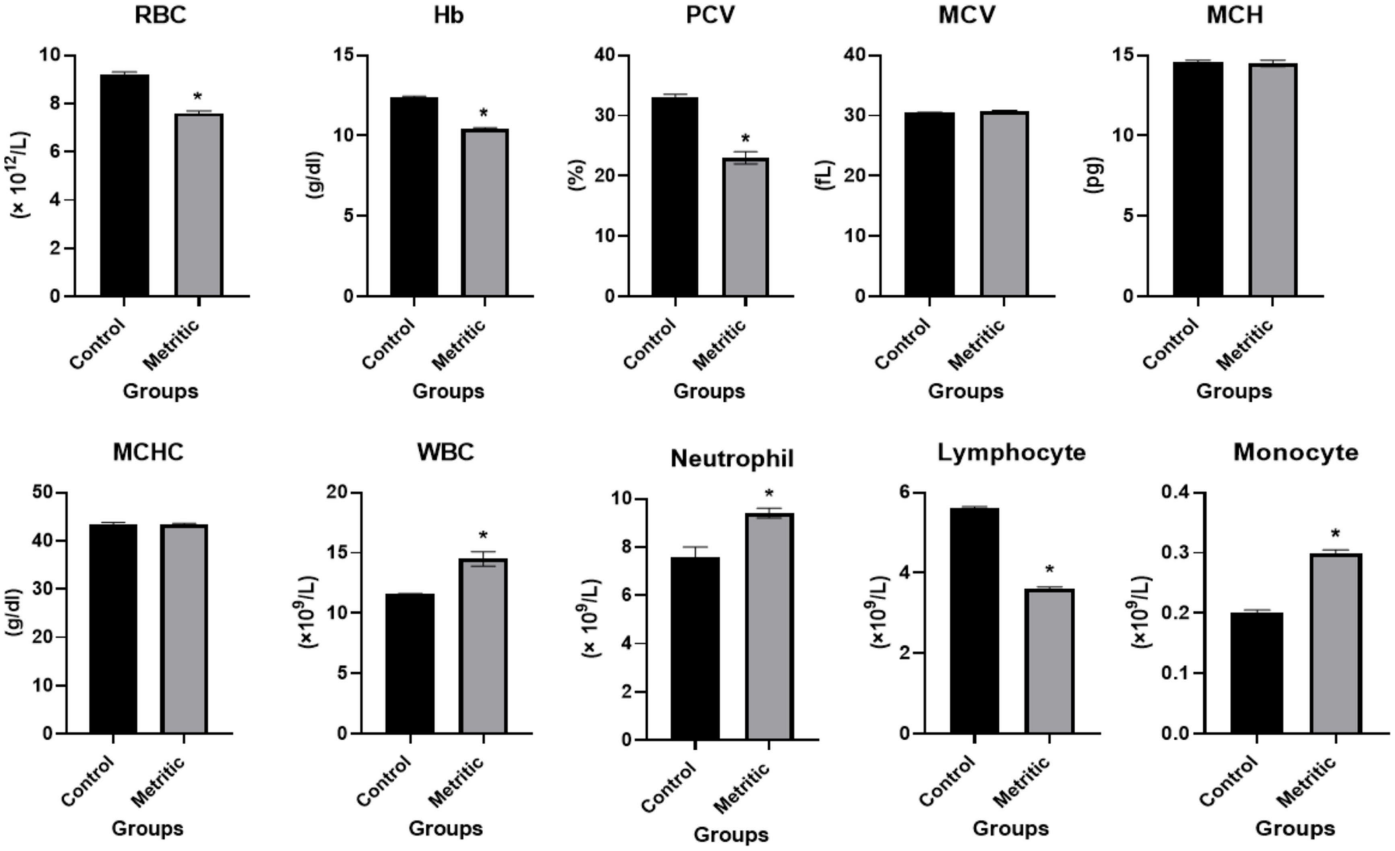
Hematological profile in control (*n*=87) and diseased group (*n*=13) she-camels. Data are expressed as mean ± standard error (SE). Asterisks (*) indicate statistically significant differences (*p* < 0.05). RBC, erythrocytes count; HB, hemoglobin; PCV: packed cell volume; MCV, mean corpuscular volume; MCH, mean corpuscular hemoglobin; MCHC, mean corpuscular hemoglobin concentration; WBC, total leukocytes count.

The present study showed significantly (*p* < 0.05) low values RBC, Hb, PCV, and lymphocyte count (7.6 ± 0.1 × 1,012/L, 10.4 ± 0.1 g/dL, 23 ± 1%, 3.6 ± 0.05 ×109/L, respectively) with a noteworthy (*p* < 0.05) increase in the values of WBC, neutrophil, and monocyte (14.5 ± 0.6, 9.4 ± 0.21, 5.6 ± 0.05, and 0.3 ± 0.005 ×109/L, respectively) in metritic she-camels compared to the healthy group (Figure 1).

### Transcriptional profiles of immune response, metabolic pathways, stress response and redox regulation, and oxidative stress defense

**Figure 2:**
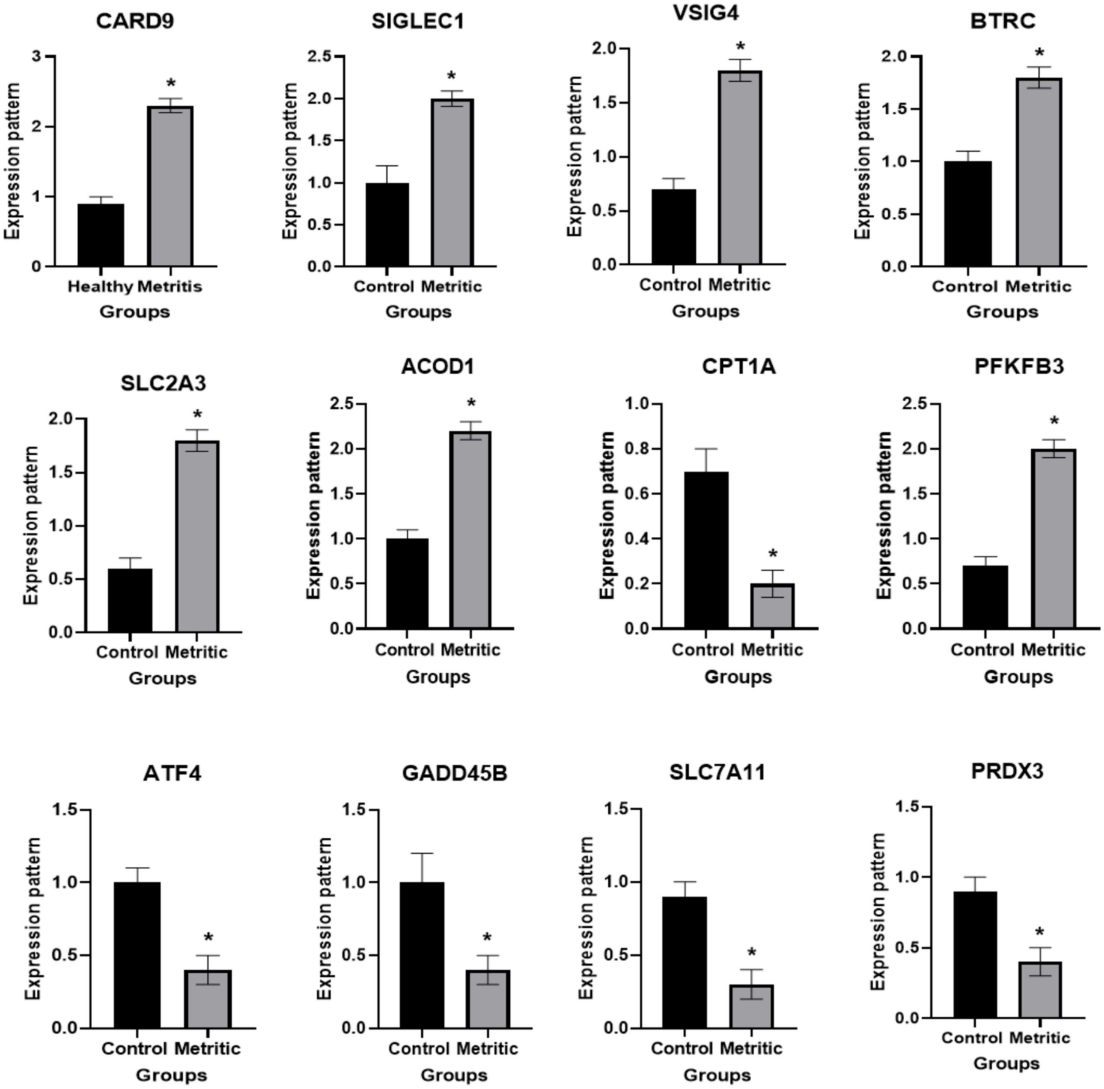
Differential transcriptional expression of immune response, metabolic pathways, stress response and redox regulation, and oxidative stress defense in healthy (*n* = 87) and diseased (*n*= 13) she camel. Data are expressed as mean ± standard error (SE). Asterisks (*) indicate statistically significant differences (*p* < 0.05). Caspase recruitment domain family member 9 (CARD9), sialic acid binding ig like lectin 1 (SIGLEC1), v-set and immunoglobulin domain containing 4 (VSIG4); beta-transducin repeat containing E3 ubiquitin protein ligase (BTRC); solute carrier family 2 member 3 (SLC2A3), aconitate decarboxylase 1 (ACOD1), carnitine palmitoyltransferase 1a (CPT1A), 6-phosphofructo-2-kinase/fructose-2,6-bisphosphatase 3 (PFKFB3); activating transcription factor 4 (ATF4), growth arrest DNA damage inducible beta (GADD45B), solute carrier family 7 member 11 (SLC7A11); and peroxiredoxin 3 (PRDX3).

Figure 2 shows the transcriptional expression patterns of the investigated genes. Compared to clinically healthy animals, *CARD9, SIGLEC1, VSIG4, BTRC, SLC2A3, ACOD1*, and *PFKFB3* showed noticeably higher expression levels in metritic she-camel. In contrast, diseased animals exhibited significant downregulation of *CPT1A, ATF4, GADD45B, SLC7A11* and *PRDX3. CARD9* showed the highest transcript abundance (2.32 ± 0.14) among metritic she-camels, whereas *CPT1A* showed the lowest expression level (0.28 ± 0.07)*. GADD45B* had the highest mRNA expression (1.05 ± 0.21), while *SLC2A3* had the lowest transcriptional level (0.68 ± 0.16) in healthy she-camels.

### Genetic polymorphism analysis

PCR amplification generated specific DNA fragments for the investigated genes, including *CARD9* (413 bp), *SIGLEC1* (339 bp), *VSIG4* (468 bp), *BTRC* (474 bp), *SLC2A3* (384 bp), *ACOD1* (383 bp), CPT1A (401 bp), *PFKFB3* (444 bp), *ATF4* (419 bp), *GADD45B* (375 bp), *SLC7A11* (445 bp), and *PRDX3* (459 bp).

Alignment with reference sequences retrieved from GenBank supported all the detected sequence variants (Supplementary Figures S1–S12). The identified polymorphisms were primarily located within exonic regions (Table 3), resulting in alterations in coding DNA sequences in metritic she-camels compared with healthy controls. Nineteen single nucleotide polymorphisms (SNPs) including nine synonymous and ten non-synonymous substitutions, were found in the genes under investigation by DNA sequencing analysis. Significant variations in SNP genotype frequencies between sick and healthy she-camels were found using statistical analysis (*p* < 0.005). When SNP distribution patterns across all genes were compared using chi-square testing, there was a significant difference between animals with and without metritis (*p* < 0.05) (Table 3).

**Table 3:** Distribution of genes related to immune response, metabolic pathways, stress response and redox regulation, and oxidative stress defense in she-camels with and without metritis, emphasizing single nucleotide changes and their anticipated genetic effects.

Gene	SNPs	Healthy *n* = 87	Metritis *n* = 13	Total *n* = 100	Chi square value X^2^	*p* value	kind of inherited change	Amino acid order and sort
*CARD9*	T132C	44/87	−/13	44/100	11.7	0.002	Synonymous	44 Y
	T251C	−/87	5/13	5/100	35.2	0.001	Non- synonymous	84 M to T
*SIGLEC1*	A181C	−/87	9/13	9/100	66.1	0.001	Non- synonymous	61 K to Q
*VSIG4*	A164G	−/87	6/13	6/100	42.7	0.001	Non- synonymous	55 N to S
	G406A	37/87	−/13	37/100	8.7	0.003	Non- synonymous	136 V to M
*BTRC*	T51A	53/87	−/13	53/100	16.8	0.001	Synonymous	17 T
	G195A	−/87	9/13	9/100	66.1	0.001	Synonymous	65 S
	A422G	48/87	−/13	48/100	13.7	0.001	Non- synonymous	141 K to R
*SLC2A3*	T63C	59/87	−/13	59/100	21.5	0.001	Synonymous	21 G
*ACOD1*	G236T	28/87	−/13	28/100	5.8	0.01	Non- synonymous	79 R to L
*CPT1A*	T108C	−/87	7/13	7/100	50.3	0.001	Synonymous	36 V
	C207T	65/87	−/13	65/100	27.7	0.001	Synonymous	69 D
	G246A	−/87	8/13	8/100	58.1	0.001	Synonymous	82 A
*PFKFB3*	A143C	−/87	11/13	11/100	82.7	0.001	Non- synonymous	48 D to A
*ATF4*	C204A	−/87	8/13	8/100	58.1	0.001	Synonymous	68 G
*GADD45B*	T90C	−/87	9/13	9/100	66.1	0.001	Synonymous	30\u00B0C
*SLC7A11*	A32G	71/87	−/13	69/100	36.5	0.001	Non- synonymous	11 N to S
	G394A	53/87	−/13	53/100	16.8	0.001	Non- synonymous	132 V to I
*PRDX3*	T174A	66/87	−/13	66/100	29	0.001	Non- synonymous	58 D to T

Caspase recruitment domain family member 9 (CARD9), sialic acid binding ig like lectin 1 (SIGLEC1), v-set and immunoglobulin domain containing 4 (VSIG4); beta-transducin repeat containing E3 ubiquitin protein ligase (BTRC); solute carrier family 2 member 3 (SLC2A3), aconitate decarboxylase 1 (ACOD1), carnitine palmitoyltransferase 1a (CPT1A), 6-phosphofructo-2-kinase/fructose-2,6-bisphosphatase 3 (PFKFB3); activating transcription factor 4 (ATF4), growth arrest DNA damage inducible beta (GADD45B), solute carrier family 7 member 11 (SLC7A11); and peroxiredoxin 3 (PRDX3).

The Hardy–Weinberg equilibrium (HWE) was evaluated separately for each SNP locus in the healthy control and metritis-affected groups using the chi-square (χ^2^) test. The results indicated that genotype frequencies at all analyzed loci were consistent with HWE in both groups, as the calculated χ^2^ values did not exceed the corresponding critical (tabulated) values (Table 4).

**Table 4:** Genotype frequencies and Hardy–Weinberg equilibrium analysis of the investigated gene loci in healthy (*n* = 87) and metritis-affected (*n* = 13) she-camels.

Gene	SNPs	Number	Total	Genotyping frequency			Allelic frequency		χ^2^
*CARD9*	T132C			TT	TC	CC	P (T)	q (C)	0.003
		Observed	100	44/0.44		56/0.56	0.25	0.75	
		Expected		6.25	37.5	56.25			
	T251C			TT	TC	CC	P (T)	q (C)	0.006
		Observed		95/0.95		5/0.05	0.78	0.22	
		Expected		60.84	34.32	4.84			
*SIGLEC1*	A181C			AA	AC	CC	P (A)	q (C)	0.0
		Observed		91/0.91		9/0.09	0.70	0.30	
		Expected		49	42	9			
*VSIG4*	A164G			AA	AG	GG	P (A)	q (G)	0.011
		Observed		94/0.94		6/0.06	0.76	0.24	
		Expected		57.76	36.48	5.76			
	G406A			GG	GA	AA	P (G)	q (A)	0.015
		Observed		37/0.37		63/0.63	0.21	0.79	
		Expected		4.41	33.18	62.41			
*BTRC*	T51A			TT	TA	AA	P (T)	q (A)	0.015
		Observed		53/0.53		47/0.47	0.31	0.69	
		Expected		9.61	42.78	47.61			
	G195A			GG	GA	AA	P (G)	q (A)	0.0
		Observed		91/0.91		9/0.09	0.70	0.30	
		Expected		49	42	9			
	A422G			AA	AG	GG	P (A)	q (G)	0.001
		Observed		48/0.48		52/0.52	0.28	0.72	
		Expected		7.84	40.32	51.84			
*SLC2A3*	T63C			TT	TC	CC	P (T)	q (C)	6.62−E05
		Observed		59/0.59		41/0.41	0.36	0.64	
		Expected		12.96	46.08	40.96			
*ACOD1*	G236T			GG	GT	TT	P (G)	q (T)	0.003
		Observed		28/0.28		72/0.72	0.15	0.85	
		Expected		2.25	25.5	72.25			
	T108C			TT	TC	CC	P (T)	q (C)	0.009
		Observed		93/0.93		7/0.07	0.74	0.26	
		Expected		54.76	38.48	6.76			
*CPT1A*	C207T			CC	CT	TT	P (C)	q (T)	0.002
		Observed		65/0.65		35/0.35	0.41	0.59	
		Expected		16.81	48.38	34.81			
	G246A			GG	GA	AA	P (G)	q (A)	0.004
		Observed		92/0.92		8/0.08	0.72	0.28	
		Expected		51.84	40.32	7.84			
*PFKKB3*	A143C			AA	AC	CC	P (A)	q (C)	0.001
		Observed		89/0.89		11/0.11	0.67	0.33	
		Expected		44.89	44.22	10.89			
*ATF4*	C204A			CC	CA	AA	P (C)	q (A)	0.004
		Observed		92/0.92		8/0.08	0.72	0.28	
		Expected		51.84	40.32	7.84			
*GADD45B*	T90C			TT	TC	CC	P (T)	q (C)	0.0
		Observed		91/0.91		9/0.09	0.70	0.30	
		Expected		49	42	9			
	A32G			AA	AG	GG	P (A)	q (G)	0.001
		Observed		71/0.71		29/0.29	0.46	0.54	
		Expected		21.16	49.68	29.16			
*SLC7A11*	G394A			GG	GA	AA	P (G)	q (A)	0.015
		Observed		53/0.53		47/0.47	0.31	0.69	
		Expected		9.61	42.78	47.61			
*PRDX3*	T174A			TT	TA	AA	P (T)	q (A)	0.006
		Observed		66/0.66		34/0.34	0.42	0.58	
		Expected		17.64	48.72	33.64			

Caspase recruitment domain family member 9 (CARD9), sialic acid binding ig like lectin 1 (SIGLEC1), v-set and immunoglobulin domain containing 4 (VSIG4); beta-transducin repeat containing E3 ubiquitin protein ligase (BTRC); solute carrier family 2 member 3 (SLC2A3), aconitate decarboxylase 1 (ACOD1), carnitine palmitoyltransferase 1a (CPT1A), 6-phosphofructo-2-kinase/fructose-2,6-bisphosphatase 3 (PFKFB3); activating transcription factor 4 (ATF4), growth arrest DNA damage inducible beta (GADD45B), solute carrier family 7 member 11 (SLC7A11); and peroxiredoxin 3 (PRDX3).

Discriminant analysis was used to evaluate the prediction potential of the identified SNP markers for categorizing health status (Table 5). The model achieved 100% classification accuracy by correctly assigning all animals to the appropriate health groups. These findings suggest the great discriminatory ability of the selected genetic markers and support their relevance in identifying inherited predisposition to metritis.

**Table 5:** Discriminant analysis is used to categorize the genes and health condition of the she-camels under study.

		Predicted group membership		Total
		Healthy	Metritis	
Count	Healthy	87	0	100
	Metritis	0	13	100
%	Healthy	87	0.0	100.0
	Metritis	0.0	13	100.0

### Biochemical and hormonal profile

Biochemically, the present study showed significantly (*p* < 0.05) low values for the serum levels of glucose, estrogen, progesterone, FSH, LH, T4, and calcium (55 ± 2.8 mg/dL, 29.3 ± 2.3 pg./mL, 0.2 ± 0.005 ng/mL, 29.3 ± 2.3 mU/mL, 1.1 ± 0.05 mU/mL, 3.7 ± 0.05 μg/dL, and 6.8 ± 0.1 mg/dL, respectively) in the metritic group compared with the healthy ones. Conversely, there was a noteworthy (*p* < 0.05) increase in the serum concentrations of NEFA, BHBA, and cortisol (0.31 ± 0.005 mmol/L, 1.4 ± 0.05 mmol/L, and 6 ± 0.05 μg/dL, respectively) in the metritic she-camel compared to the healthy group (Table 6).

**Table 6:** Biochemical and hormonal profile in control (*n* = 87) and diseased group (*n* = 13) she-camels.

Variables	Control animals	Diseased animals	*P*-value
Glucose (mg/dL)	95.6 ± 3.4	55 ± 2.8*	0.001
NEFA (mmol/L)	0.21 ± 0.005	0.31 ± 0.005*	0.001
BHBA (mmol/L)	0.6 ± 0.005	1.4 ± 0.005*	0.001
Calcium(mg/dL)	8.5 ± 0.2	6.8 ± 0.1*	0.001
Estrogen (pg/mL)	45 ± 2.8	29.3 ± 2.3*	0.01
Progesterone (ng/mL)	0.4 ± 0.005	0.2 ± 0.005*	0.001
FSH (mU/mL)	3.6 ± 0.08	29.3 ± 2.3*	0.002
LH (mU/mL)	2.3 ± 0.06	1.1 ± 0.05*	0.001
Cortisol (ug/dL)	3.9 ± 0.05	6 ± 0.05*	0.001
T4 (μg/dL)	5.9 ± 0.05	3.7 ± 0.05*	0.001

Data are expressed as mean ± standard error (SE). NEFA, Non-Esterified Fatty Acids; BHBA, Betahydroxy-butyric acid; FSH, Follicle-Stimulating Hormone; LH, Luteinizing Hormone; T4, Thyroxine. *Statistically significant when *p*<0.05.

### Immunological and antioxidants profile

Regarding changes in cytokines, the serum levels of IL1-*α*, IL1-*β*, IL-6, IL-10, and TNF-α were significantly (P<0.05) increased in metritic she-camel compared to healthy ones. With respect to antioxidant/oxidative stress biomarkers, CAT, GSH, and TAC showed a significant decrease associated with a significant increase in MDA in metritic she-camel compared to healthy group (Table 7).

**Table 7:** Mean values (Mean ± SE) of immunological and antioxidant parameters of healthy (*n* = 87) and diseased (*n* = 13) she camel.

Parameters	Healthy animals	Diseased animals	*P*-value
IL1α (pg/ml)	44.3 ± 4.3	162.7 ± 7.7*	0.001
IL 1β (pg/ml)	41.6 ± 1.8	183.8 ± 6.8*	0.001
IL 6 (pg/ml)	44.2 ± 2	140.4 ± 1.3*	0.001
IL10 (pg/ml)	49.4 ± 2.7	211 ± 1.5*	0.001
TNFα (pg/mL)	43.6 ± 4.6	163.8 ± 1.8*	0.001
CAT (U/mL)	40 ± 0.5	25 ± 0.5*	0.001
GSH (mg/dl)	38 ± 0.5	22 ± 0.5*	0.001
TAC (mM/L)	55 ± 0.5	27.3 ± 0.8*	0.001
MDA (nmol / mL)	5.9 ± 0.05	17.5 ± 0.05*	0.001

IL1-α, Interleukin 1 alpha; IL1-β, Interleukin 1 beta; IL6, Interleukin 6; TNF-α, Tumor necrosis factor-alpha; IL10, Interleukin 10; GSH, Glutathione reductase; CAT, Catalase; TAC, Total antioxidant capacity dismutase; MDA, Malondialdhyde. Statistically significant between control group and PT is indicated by (*), when *p* < 0.05.

### Correlation between gene expression pattern and serum profile of hormonal, oxidative, and immunological markers in metritic she camel

Correlation analysis between gene expression patterns and serum hormonal, oxidative, and immunological markers in metritic she-camels revealed several significant associations between the two. The mRNA expression level of *CARD9* showed a strong positive correlation with serum FSH concentration (r = 0.997, *p* = 0.04). In contrast, *VSIG4* expression was negatively correlated with catalase (CAT) activity and cortisol levels (r = −0.999, *p* = 0.03). A negative correlation was observed between *BTRC* mRNA expression and circulating estrogen levels (r = −0.1, *p* = 0.009). Similarly, *ACOD1* expression was negatively associated with CAT activity and cortisol levels (*r* = −0.1, *p* = 0.008). Conversely, *PFKFB3* mRNA expression was strongly positive correlated with luteinizing hormone (LH) levels (r = 0.999, *p* = 0.02). The expression of *ATF4* was negatively correlated with IL-1α, IL-10, and FSH levels (r = −0.1, *p* = 0.001; r = −0.998, *p* = 0.03; and *r* = −0.997, *p* = 0.04, respectively). Furthermore, *GADD45B* mRNA expression showed a strong negative correlation with IL-6 levels (r = −0.999, p = 0.02), whereas *PRDX3* expression was negatively associated with total antioxidant capacity (TAC) (r = −0.1, *p* = 0.01).

A schematic summary illustrating the proposed pathophysiological mechanism underlying postpartum metritis in she-camels is presented in Supplementary Figure S13. The schematic integrates the major findings of the present study, demonstrating the relationships between negative energy balance, endocrine and metabolic alterations, oxidative stress, inflammatory cytokine production, dysregulated immune responses, changes in antioxidant- and inflammation-related gene expression, and the subsequent development and progression of postpartum metritis. The figure provides a comprehensive overview of the interconnected pathways identified in the present investigation and summarizes the proposed mechanisms contributing to the pathogenesis of metritis in postpartum she-camels.

## Discussion

The present study provides a novel integrative framework linking transcriptional dysregulation with genetic polymorphisms in metritic she-camels. While previous studies in livestock have typically examined either gene expression or SNP variation independently, the current study combines both layers of molecular information, offering a more comprehensive understanding of disease susceptibility and progression. This integrative approach represents a key advancement in camel reproductive immunogenomics, a field that remains relatively underexplored compared to cattle and small ruminants. Clinically, metritic she-camels displayed fever, increased pulse and respiratory rates, and abnormal uterine discharge. Similar manifestations have been documented in other studies (53–56).

The upregulation of immune-related genes (*CARD9, SIGLEC1*, and *VSIG4*) supports the strong activation of innate immune pathways in response to uterine infection. This finding aligns with studies in dairy cattle, where inflammatory mediators and pattern recognition receptors are significantly elevated during metritis and endometritis (57, 58). However, the novelty of the present study may indicate the involvement of CARD9-associated signaling pathways in camels, which has not been previously reported in this species in the context of uterine disease. Given the central role of *CARD9* in antifungal and antibacterial immunity, its marked upregulation suggests a possible role in camel pathogen-specific immune responses.

Similarly, increased *SIGLEC1* expression reflects macrophage activation and antigen presentation, consistent with the findings of bovine uterine infections (59). The concurrent upregulation of *VSIG4,* an immune checkpoint regulator, suggests a tightly controlled inflammatory response. The simultaneous activation of pro-inflammatory and regulatory immune genes represents a novel observation that may indicate that camel metritis involves both immune stimulation and modulation to prevent excessive tissue damage.

The elevated expression of *BTRC* highlights activation of ubiquitin-mediated signaling pathways, particularly those regulating NF-κB activity. Although ubiquitination pathways have been implicated in bovine endometritis (60), their involvement in camel metritis has not been previously documented. Thus, the present findings provide new evidence linking proteasomal regulation to uterine inflammation in camels.

Metabolic reprogramming was evident through the upregulation of SLC2A3, ACOD1, and *PFKFB3*, which may indicate a shift toward glycolysis. This metabolic shift is well established in activated immune cells and has been described in cattle with uterine diseases (61). However, the identification of *ACOD1* upregulation in camel metritis is particularly novel, as this gene encodes itaconate, a key immunometabolite with antimicrobial and anti-inflammatory activities. This suggests that camel immune cells may employ immunometabolic defense strategies similar to those in other species.

In contrast, the downregulation of *CPT1A* may indicate the suppression of mitochondrial fatty acid oxidation, reinforcing the shift toward glycolytic metabolism. This finding is consistent with the metabolic dysfunction reported in inflammatory diseases in cattle (62). The combined observation of enhanced glycolysis and suppressed lipid oxidation provides new insights into the energy metabolism imbalance in camel metritis, which has not been previously characterized.

The reduced expression of stress-response genes (*ATF4* and *GADD45B*) suggests impaired cellular adaptation to inflammatory stress. While these genes have been studied in general inflammatory conditions (63), their coordinated downregulation in camel metritis represents a novel indication of stress-response exhaustion in uterine tissue.

Similarly, the downregulation of *SLC7A11* and *PRDX3* highlights compromised antioxidant defense and increased oxidative stress. Oxidative imbalance has been widely reported in mastitis and endometritis in cattle and goats (64), but the specific involvement of the mitochondrial antioxidant gene *PRDX3* in camel metritis is described here for the first time, suggesting that mitochondrial dysfunction is a key contributor to disease pathology.

From the SNP analysis, the identification of 19 polymorphisms, including non-synonymous variants, provides strong evidence of a genetic predisposition to metritis. Previous studies in cattle have identified SNPs in immune-related genes, such as *TLR4, CXCR1*, and lactoferrin, associated with uterine disease susceptibility (65, 66). However, this study is among the first to report SNPs in *CARD9, ACOD1*, and oxidative stress-related genes in camels, highlighting species-specific genetic markers that may influence the resistance to disease.

Comparative sequence analysis revealed that many of the SNPs identified in dromedary camels occurred at evolutionarily conserved nucleotide positions shared with other camelid and ruminant species. Specifically, the CARD9 SNPs T132C and T251C corresponded to nucleotides reported in domestic water buffalo (XM_006062202.4), whereas the SIGLEC1 SNP A181C matched homologous positions in sheep (XM_042229757.1) and cattle (XM_027559353.1). The identified VSIG4 polymorphism was also shared by buffalo (XM_025275787.2) and sheep (XM_012106592.4). In BTRC, the SNPs T51A and G195A corresponded to the positions reported in buffalo (XM_006053167.3), whereas A422G was observed at homologous sites in sheep (XM_042238876.2) and cattle (XM_005225572.4). Likewise, the SLC2A3 SNP T63C and ACOD1 SNP G236T were identical to the nucleotides reported in *Camelus bactrianus* (XM_074357820.1 and XM_010973537.3, respectively). For CPT1A, SNP T108C was shared with *Camelus ferus* (XM_032490055.1) and cattle (XM_059875504.1), C207T corresponded to a nucleotide reported in *Camelus bactrianus* (XM_074371585.1), and G246A matched a homologous position in *Camelus ferus* (XM_032490055.1). The PFKFB3 SNP A143C was retained in sheep (XR_011258170.1) and *Camelus ferus* (XM_032473949.1), whereas the ATF4 SNP C204A corresponded to a homologous site in sheep (XR_011258170.1). Similarly, the GADD45B SNP T90C was maintained across goats (XM_018050778.1), sheep (XM_004008628.5), and cattle (XM_019964525.2), whereas SLC7A11 SNPs A32G and G394A were shared with wild banteng (*Bos javanicus*; XM_061384179.1), goats (XM_005691243.3), and domestic water buffalo (XM_055550993.1). Finally, the PRDX3 SNP T174A corresponded to a nucleotide reported in cattle (GenBank accession number: NM_174432.2). The occurrence of these polymorphisms at evolutionarily preserved positions across phylogenetically related species suggests that these genomic regions have been retained during evolution because of their functional significance. Evolutionary conservation is generally considered an indicator of selective pressure to maintain biological functions, particularly in genes involved in immune regulation, inflammatory signaling, cellular metabolism, stress adaptation, and antioxidant defense. Therefore, the retention of these nucleotide positions across multiple species supports the potential biological significance of the identified variants and suggests that they may contribute to genetic variation in host responses to uterine inflammation and susceptibility to metritis in dromedary camels. Similar observations have been reported in livestock species, where polymorphisms within evolutionarily conserved regions of immune and metabolic genes have been associated with variations in disease resistance, inflammatory responses, and adaptation to physiological stress (57, 67, 68).

The present study demonstrated that both synonymous and non-synonymous SNPs were significantly associated with the incidence of metritis in dromedary she-camels. Most of the non-synonymous variants were detected exclusively or at a markedly higher frequency in metritis-affected animals, suggesting that these polymorphisms may contribute to susceptibility to disease. These mutations resulted in amino acid substitutions involving changes in side-chain characteristics, including alterations in charge (K → Q, R → L, D → A, D → T), polarity (M → T, N → S), and hydrophobicity (V → M, V → I), which may influence protein conformation, stability, receptor binding, enzymatic activity, and protein–protein interactions. Such structural alterations could impair the normal functions of proteins involved in innate immune signaling (CARD9, SIGLEC1, VSIG4, and BTRC), immunometabolism (SLC2A3, ACOD1, CPT1A, and PFKFB3), and antioxidant defense (SLC7A11 and PRDX3), thereby weakening the uterine immune responses and increasing susceptibility to metritis. Although synonymous SNPs do not alter the amino acid sequence, accumulating evidence indicates that they can affect mRNA stability, splicing efficiency, translational kinetics, and protein expression, thereby contributing to phenotypic variation (69, 70). Collectively, these findings suggest that the identified SNPs represent promising molecular markers associated with metritis susceptibility in dromedary camels; however, further functional studies are warranted to validate their biological effects and diagnostic potential in other populations.

A particularly important novel finding is the complete discrimination (100% accuracy) between healthy and metritic she-camel based on SNP profiles. Although this may partly reflect the sample size, it is consistent with the potential contribution of the selected markers. This level of classification performance has rarely been reported for livestock reproductive diseases, suggesting that these markers may represent promising candidates for future studies investigating genetic susceptibility and biomarker-assisted diagnosis in camel populations.

Animals with metritis showed pronounced alterations in hematological parameters, reflecting both the systemic inflammatory response and the detrimental effects of uterine infection on hematopoiesis. In the present study, metritic she-camels showed a significant reduction in erythrogram indices, including red blood cell (RBC) count, hemoglobin (Hb) concentration, and packed cell volume (PCV). These findings are indicative of normocytic normochromic anemia, a characteristic feature of anemia associated with inflammatory and infectious diseases. This form of anemia is largely mediated by pro-inflammatory cytokines, such as interleukin-1 and tumor necrosis factor-*α*, which inhibit erythropoietin production, disrupt iron homeostasis, and ultimately suppress erythrocyte synthesis (71).

The leukogram findings further corroborate the presence of an active inflammatory process in metritic she-camel. A significant increase in the total leukocyte count, mainly attributed to neutrophilia, was observed, reflecting the innate immune response to bacterial invasion of the uterus. This neutrophilic response is often accompanied by a left shift, suggesting increased bone marrow activity and the release of immature neutrophils into the circulation (72). Additionally, lymphopenia may occur, likely due to stress-induced corticosteroid release, leading to lymphocyte redistribution (73). Monocytosis can be interpreted as a response to ongoing tissue damage and inflammation, as monocytes play a crucial role in phagocytosis and tissue repair during the chronic phase of infection. These hematological alterations are in agreement with previous studies that reported similar changes in animals affected by metritis and other uterine infections (74–77). Collectively, the hematological profile of metritic she-camel reflects a state of systemic inflammation, immune activation, and chronic disease-related anemia. Such changes not only provide insight into the underlying pathophysiology of metritis but also serve as valuable diagnostic and prognostic indicators for evaluating disease severity and monitoring the effectiveness of therapeutic interventions in dairy herds.

Negative energy balance (NEB) during the postpartum period disrupts innate immune function and predisposes animals to clinical metritis. In the present study, metritic she-camels exhibited significantly lower blood glucose levels alongside elevated serum NEFA and BHBA concentrations, supporting their value as metabolic biomarkers of the disease. These findings agree with previous reports suggesting a negative association between glucose levels and the incidence of metritis, as well as persistent uterine infections (78, 79). Hypoglycemia, resulting from reduced feed intake and increased metabolic demands during late pregnancy and early lactation, promotes lipolysis and mobilization of adipose tissue reserves, leading to increased circulating NEFA and BHBA (80). Elevated NEFA levels induce lipotoxic effects, impair ovarian function, and reduce neutrophil activity, thereby compromising immune defense and facilitating uterine infection (81–85). Similarly, increased BHBA reflects enhanced ketogenesis and is strongly associated with a higher risk of postpartum disorders. These metabolic alterations may indicate adaptation to lactation and sustained NEB, which contribute to immune dysfunction and persistence of uterine infections (53–55). Furthermore, decreased glucose availability negatively affects immune cell proliferation and function, increasing susceptibility to infection (86). In ruminants, reduced glucose levels during the postpartum period are primarily linked to increased demands for milk synthesis and may be exacerbated by impaired hepatic gluconeogenesis during systemic inflammation (53, 87). In addition, calcium concentrations were significantly lower in metritic she-camel, supporting the association between hypocalcemia and postpartum uterine diseases (56, 78, 88, 89). Reduced calcium levels impair neutrophil function and uterine contractility, leading to delayed uterine involution and increased bacterial persistence (89–92). Collectively, these metabolic and mineral imbalances play a possible role in the pathogenesis of metritis and its associated reproductive dysfunction.

The endocrinological profile revealed that metritic she-camels exhibited significantly higher cortisol levels, along with reduced concentrations of FSH, LH, E2, P4, and T4 compared to healthy controls, which is consistent with previous reports (56, 76, 93, 94). The resumption of postpartum ovarian cyclicity depends on normal hypothalamic–pituitary function and the structural and functional integrity of the reproductive tract. In dromedary camels, the specialized histological organization of the uterotubal junction highlights the close relationship between reproductive tract morphology and successful reproductive function, suggesting that postpartum uterine inflammation may adversely affect reproductive efficiency beyond the uterus (95). Although FSH levels may remain relatively unchanged and early follicular waves can occur after parturition, uterine infection impairs reproductive function primarily by suppressing GnRH and LH secretion. Endotoxins, particularly those from *Escherichia coli*, reduce pituitary responsiveness to GnRH, thereby inhibiting dominant follicle ovulation (96). Furthermore, uterine bacterial load and associated inflammation disrupt follicular growth and ovarian activity by decreasing LH pulse frequency, a condition exacerbated by a negative energy balance (97). This leads to a reduced follicular size, lower estradiol secretion, and impaired luteal development. Normal ovarian follicular dynamics and morphometric characteristics are essential for adequate steroidogenesis and fertility in dromedary camels, and disruption of these processes during uterine disease may further compromise reproductive performance (98). Consequently, affected animals exhibit smaller corpora lutea and decreased progesterone concentrations compared to healthy animals (99). Recent metabolomic analyses of dromedary camel follicular fluid have further demonstrated that ovarian metabolism is highly dynamic and closely associated with reproductive status, with significant alterations in metabolites involved in energy metabolism, oxidative balance, and follicular function (100). These findings support the present results, suggesting that metabolic disturbances accompanying postpartum metritis may extend beyond systemic energy imbalance to influence the ovarian microenvironment, thereby contributing to impaired follicular development and reduced endocrine activity.

In the present study, metritic she-camels exhibited a disturbed oxidative balance, as indicated by significantly increased MDA levels and decreased CAT, GSH, and TAC activities compared with healthy animals. These findings are consistent with previous reports linking uterine inflammation to enhanced oxidative stress and weakened antioxidant defense (36, 101). Elevated reactive oxygen species (ROS) levels during infection promote lipid peroxidation and cellular damage, as reflected by increased MDA levels, while the reduction in antioxidant enzymes suggests their consumption in counteracting oxidative stress.

Comparative studies among domestic ruminants have indicated that oxidative stress is a common pathological mechanism underlying postpartum uterine diseases, although the magnitude of these alterations differs among species (34, 102). In dairy cattle, metritis is consistently associated with increased ROS generation, enhanced lipid peroxidation, depletion of antioxidant defenses, and excessive production of inflammatory mediators in both blood and uterine tissues (30, 33, 103, 104). Similar alterations have also been described in sheep with postpartum uterine inflammation, where increased oxidative stress and impaired antioxidant status are associated with delayed uterine recovery and reduced reproductive performance. These findings suggest that redox imbalance represents a conserved mechanism in the pathogenesis of postpartum uterine disease across ruminant species, although species-specific differences in immune responses and antioxidant capacity may influence the severity of the disease. Therefore, evaluating oxidative stress biomarkers alongside inflammatory mediators may improve the understanding of metritis pathogenesis and facilitate the identification of reliable diagnostic and prognostic indicators of metritis.

Moreover, metritic she-camel showed increased levels of cytokines (IL-1*α*, IL-1β, IL-6, IL-10, and TNF-α), highlighting the close interaction between oxidative stress and inflammation. This response is triggered by pathogen-associated molecular patterns, such as lipopolysaccharides from Gram-negative bacteria, which are recognized by pattern recognition receptors, including Toll-like receptors on immune and endometrial cells. Activation of downstream signaling pathways, particularly NF-κB and MAPK, leads to enhanced cytokine production, promoting immune cell recruitment and inflammatory responses necessary for pathogen clearance (6, 105, 106). These findings are in agreement with previous studies reporting similar oxidative and inflammatory alterations in animals affected by postpartum uterine diseases (36, 107, 108).

The observed correlations between gene expression patterns and serum hormonal, oxidative, and immunological markers in metritic she-camels provide insights into the complex pathophysiology of uterine inflammation. The strong positive association between CARD9 expression and FSH levels suggests a potential interaction between immune signaling and reproductive endocrine regulation. This is supported by recent evidence indicating that CARD9 acts as a key adaptor in innate immunity, regulating cytokine production and inflammatory responses through NF-κB and MAPK pathways (106). Moreover, CARD9-mediated signaling is linked to oxidative stress modulation, further supporting its role in coordinating immune and metabolic responses during infection (109). In contrast, the negative correlations between VSIG4 and ACOD1 with catalase activity and cortisol levels may reflect immune dysregulation associated with oxidative imbalance and stress hormone alterations. Notably, ACOD1 (IRG1) has been identified as a key regulator of immunometabolism, producing itaconate to modulate inflammatory and oxidative pathways during infections (110). These findings suggest that increased inflammatory signaling may suppress antioxidant defenses, contributing to oxidative stress in animals with metritis. Collectively, these results support the concept that metritis involves integrated disturbances in immune signaling, oxidative balance, and endocrine regulation, where key genes, such as CARD9 and ACOD1 act as central mediators linking inflammation with metabolic and hormonal alterations.

The present study had several limitations that should be considered when interpreting the findings. First, although all eligible postpartum she-camels available during the study period were enrolled, only 13 animals developed clinical metritis, resulting in an imbalanced distribution between the metritic and healthy groups in the study. This reflects the natural occurrence of the disease under field conditions but may limit the statistical power and generalizability of these findings. Second, the observational design of the study allowed the identification of associations between the investigated biomarkers and clinical metritis but did not establish causal relationships or predictive values. Third, bacteriological culture and pathogen identification were not performed because the primary objective was to characterize the host hematological, biochemical, hormonal, oxidative stress, inflammatory, and molecular responses associated with naturally occurring clinical metritis rather than to determine its microbial etiology. Finally, although the identified biomarkers and classification model showed promising discriminatory performance within the present dataset, they have not been validated externally. Moreover, while the study focused on statistical significance and the strength of associations, additional standardized effect size measures, such as Cohen’s d for group comparisons, would provide complementary information regarding the magnitude of the observed differences and further strengthen the interpretation of the findings, particularly in studies with larger and more balanced sample sizes. Therefore, larger multicenter prospective studies, including a greater number of metritic camels, bacteriological characterization, independent validation cohorts, and comprehensive effect size reporting, are required before these biomarkers can be considered for diagnostic, prognostic, or marker-assisted breeding applications.

## Conclusion

This study identified significant hematological, metabolic, hormonal, inflammatory, oxidative stress, transcriptional, and genetic differences between healthy and metritic dromedary she-camels. The findings suggest that alterations in immune-, metabolic-, stress response-, and antioxidant-related genes are associated with metritis and may contribute to disease susceptibility. The identified biomarkers and SNPs are promising candidates for future validation studies. However, larger cohorts and independent populations are required before their application in diagnostic programs or genetic selection strategies can be recommended.

## Data Availability

The original contributions presented in the study are included in the article/Supplementary material, further inquiries can be directed to the corresponding authors.

## References

[1] Suliman GM Alowaimer AN Hussein EO Ali HS Abdelnour SA El-Hack MEA . Chemical composition and quality characteristics of meat in three one-humped camel (*Camelus dromedarius*) breeds as affected by muscle type and post-mortem storage period. Animals. (2019) 9:834. doi: 10.3390/ani9100834, 31635171 PMC6826607

[2] Swelum AA-A Saadeldin IM Abdelnour SA Ba-Awadh H Abd El-Hack ME Sheiha AM. Relationship between concentrations of macro and trace elements in serum and follicular, oviductal, and uterine fluids of the dromedary camel (*Camelus dromedarius*). Trop Anim Health Prod. (2020) 52:1315–24. doi: 10.1007/s11250-019-02137-0, 31760562

[3] Sghiri A Ciccarelli M Waqas MS Anouassi A Tibary A. Advances in the diagnosis of reproductive disorders in female camelids. Animals. (2025) 15:2902. doi: 10.3390/ani15192902, 41096497 PMC12523362

[4] Faraz A Waheed A Mirza R Ishaq H Tariq M. Socio economic status and associated constraints of camel production in desert Thal Punjab. Pakistan J Fish Livest Prod. (2019) 7:288. doi: 10.4172/2332-2608.1000288

[5] Tibary A El Allali K. Dromedary camel: a model of heat resistant livestock animal. Theriogenology. (2020) 154:203–11. doi: 10.1016/j.theriogenology.2020.05.046, 32663620

[6] Várhidi Z Csikó G Bajcsy ÁC Jurkovich V. Uterine disease in dairy cows: a comprehensive review highlighting new research areas. Vet Sci. (2024) 11:66. doi: 10.3390/vetsci11020066, 38393084 PMC10893454

[7] Sheldon IM Lewis GS LeBlanc S Gilbert RO. Defining postpartum uterine disease in cattle. Theriogenology. (2006) 65:1516–30. doi: 10.1016/j.theriogenology.2005.08.021, 16226305

[8] Gherissi DE Lamraoui R Chacha F Bouzebda Z Bouzebda FA Hanzen C. Genital abnormalities associated to lack of uterine adenogenesis or endometrial gland dysgenesis of female dromedary camels (*Camelus dromedarius*). Open Vet J. (2020) 10:44–52. doi: 10.4314/ovj.v10i1.8, 32426256 PMC7193880

[9] Ali A Derar DR Almundarij TI. Aetiological analysis and diagnosis of reproductive disorders in male dromedary camels. Reprod Domest Anim. (2021) 56:1267–73. doi: 10.1111/rda.13988, 34219309

[10] Qureshi Z Muhammad G Athar M Lodhi L. Acute puerperal metritis in a dromedary camel (*Camelus dromedarius*). Pak Vet J. (2002) 22:45–7.

[11] Underwood S Bathgate R Maxwell W Evans G. Birth of offspring after artificial insemination of heifers with frozen-thawed, sex-sorted, re-frozen-thawed bull sperm. Anim Reprod Sci. (2010) 118:171–5. doi: 10.1016/j.anireprosci.2009.08.007, 19765921

[12] Hanzen C. La Propédeutique de l’appareil génital Femelle Des Ruminants. Liège, Belgium: Université de Liège (ULiège) (2017).

[13] Ali A Derar D Alsamri A Al Sobayil F. Echography of clinically relevant disorders in the genital tract of female dromedary camels. Anim Reprod Sci. (2017) 182:123–33. doi: 10.1016/j.anireprosci.2017.05.007 28545987

[14] Barlund C Carruthers T Waldner C Palmer C. A comparison of diagnostic techniques for postpartum endometritis in dairy cattle. Theriogenology. (2008) 69:714–23. doi: 10.1016/j.theriogenology.2007.12.005, 18242670

[15] Azawi O. Postpartum uterine infection in cattle. Anim Reprod Sci. (2008) 105:187–208. doi: 10.1016/j.anireprosci.2008.01.010, 18280065

[16] Sordillo L. Nutritional strategies to optimize dairy cattle immunity. J Dairy Sci. (2016) 99:4967–82. doi: 10.3168/jds.2015-10354, 26830740

[17] Senosy W Izaike Y Osawa T. Influences of metabolic traits on subclinical endometritis at different intervals postpartum in high milking cows. Reprod Domest Anim. (2012) 47:666–74. doi: 10.1111/j.1439-0531.2011.01941.x, 22053752

[18] Hussen J Shawaf T Al-Mubarak AI Al Humam NA Almathen F Schuberth H-J. Leukocyte populations in peripheral blood of dromedary camels with clinical endometritis. Anim Reprod Sci. (2020) 222:106602. doi: 10.1016/j.anireprosci.2020.106602 32980651

[19] Shehab-El-Deen M Al-Dobaib S Al-Sobayil K. Metabolic changes in the blood of dromedary camel at early post-partum. J Agric Sci. (2020) 12:21–30. doi: 10.5539/jas.v12n1p21

[20] Al Ramadan SY Salem KTA-M Alshubaith IH Al-Ali AM Abohelaika S Moqbel MS . Selected aspects of camel immune system and immune responses. Open J Vet Med. (2021) 11:177–211. doi: 10.4236/ojvm.2021.116013

[21] Pohl A Burfeind O Heuwieser W. The associations between postpartum serum haptoglobin concentration and metabolic status, calving difficulties, retained fetal membranes, and metritis. J Dairy Sci. (2015) 98:4544–51. doi: 10.3168/jds.2014-9181, 25912860

[22] Rodríguez E Arís A Bach A. Associations between subclinical hypocalcemia and postparturient diseases in dairy cows. J Dairy Sci. (2017) 100:7427–34. doi: 10.3168/jds.2016-12210, 28690056

[23] Ali A Tharwat M Al-Sobayil F. Hormonal, biochemical, and hematological profiles in female camels (*Camelus dromedarius*) affected with reproductive disorders. Anim Reprod Sci. (2010) 118:372–6. doi: 10.1016/j.anireprosci.2009.08.014, 19815355

[24] Icalho M Marques E Gilbert R Bicalho R. The association of plasma glucose, BHBA, and NEFA with postpartum uterine diseases, fertility, and milk production of Holstein dairy cows. Theriogenology. (2017) 88:270–82. doi: 10.1016/j.theriogenology.2016.09.036 27793454

[25] Chapinal N Carson M Duffield T Capel M Godden S Overton M . The association of serum metabolites with clinical disease during the transition period. J Dairy Sci. (2011) 94:4897–903. doi: 10.3168/jds.2010-4075, 21943741

[26] Ospina P Nydam D Stokol T Overton T. Evaluation of nonesterified fatty acids and β-hydroxybutyrate in transition dairy cattle in the northeastern United States: critical thresholds for prediction of clinical diseases. J Dairy Sci. (2010) 93:546–54. doi: 10.3168/jds.2009-2277, 20105526

[27] Surai PF Earle-Payne K. Antioxidant defences and redox homeostasis in animals. Antioxidants. (2022) 11:1012. doi: 10.3390/antiox11051012 35624875 PMC9137460

[28] Koshevoy V Naumenko S Sklyarov P Sklyarov P Sklyarov P Skliarov P . Male infertility: pathogenetic significance of oxidative stress and antioxidant defence. Sci Horiz. (2021) 24:107–16. doi: 10.48077/scihor.24(6).2021.107-116

[29] Mohamed RH Khalphallah A Nakada K Elmeligy E Hassan D Ebissy EA . Clinical and correlated responses among steroid hormones and oxidant/antioxidant biomarkers in pregnant, non-pregnant and lactating CIDR-pre-synchronized dromedaries (*Camelus dromedarius*). Vet Sci. (2021) 8:247. doi: 10.3390/vetsci8110247, 34822620 PMC8624123

[30] Boni R Cecchini Gualandi S. Relationship between oxidative stress and endometritis: exploiting knowledge gained in mares and cows. Animals. (2022) 12:2403. doi: 10.3390/ani12182403, 36139263 PMC9495037

[31] Zachut M Contreras GA. Symposium review: mechanistic insights into adipose tissue inflammation and oxidative stress in periparturient dairy cows. J Dairy Sci. (2022) 105:3670–86. doi: 10.3168/jds.2021-21225, 35151484

[32] Ayemele AG Tilahun M Lingling S Elsaadawy SA Guo Z Zhao G . Oxidative stress in dairy cows: insights into the mechanistic mode of actions and mitigating strategies. Antioxidants. (2021) 10:1918. doi: 10.3390/antiox10121918, 34943022 PMC8750585

[33] Malledevarahalli Chandrappa S Pascottini OB Opsomer G Meineri G Martino NA Banchi P . Circulating and endometrial cell oxidative stress in dairy cows diagnosed with metritis. Theriogenology. (2023) 198:217–23. doi: 10.1016/j.theriogenology.2022.12.045, 36610371

[34] Koshevoy V Naumenko S Skliarov P Syniahovska K Vikulina G Klochkov V . Effect of gadolinium orthovanadate nanoparticles on male rabbits’ reproductive performance under oxidative stress. World's Vet J. (2022) 12:296–303. doi: 10.54203/scil.2022.wvj37

[35] Darwish A Ebissy E Hafez A Ateya A El-Sayed A. Nucleotide sequence variants, gene expression and serum profile of immune and antioxidant markers associated with bacterial diarrhea susceptibility in Barki lambs. BMC Vet Res. (2024) 20:462. doi: 10.1186/s12917-024-04288-1, 39394128 PMC11468138

[36] El-Sayed A Refaai M Ateya A. Doppler ultrasonographic scan, gene expression and serum profile of immune, APPs and antioxidant markers in Egyptian buffalo–cows with clinical endometritis. Sci Rep. (2024) 14:5698. doi: 10.1038/s41598-024-56258-0, 38459095 PMC10923904

[37] Sayed AE Hafez A Ateya A Darwish A Tahoun A. Single nucleotide polymorphisms, gene expression and evaluation of immunological, antioxidant, and pathological parameters associated with bacterial pneumonia in Barki sheep. Ir Vet J. (2025) 78:11. doi: 10.1186/s13620-025-00296-1, 40221769 PMC11992743

[38] Berry DP Bermingham ML Good M More SJ. Genetics of animal health and disease in cattle. Ir Vet J. (2011) 64:5. doi: 10.1186/2046-0481-64-5, 21777492 PMC3102331

[39] Nicholas FW. Animal breeding and disease. Philos Trans Royal Soc B Biol Sci. (2005) 360:1529–36. doi: 10.1098/rstb.2005.1674, 16048793 PMC1569506

[40] Mansouri A Akthar I Miyamoto A. TLR2 and TLR4 bridge physiological and pathological inflammation in the reproductive system. Commun Biol. (2025) 8:1008. doi: 10.1038/s42003-025-08424-x, 40618011 PMC12228841

[41] Nash DM Giles JL. Uterine inflammation and lessons from large animal models of endometritis. Nat Rev Immunol. (2025) 25:934–46. doi: 10.1038/s41577-025-01200-2, 40696155

[42] Askar AR Masoud A El-Bordeny NE Kewan KZ El-Galil ERA El Ezz SSA . Grazing camels under semi-extensive production system: selectivity, feed intake capacity, digestion and energy expenditure. BMC Vet Res. (2024) 20:364. doi: 10.1186/s12917-024-04199-1, 39138422 PMC11321051

[43] Gautam A Donohue D Hoke A Miller SA Srinivasan S Sowe B . Investigating gene expression profiles of whole blood and peripheral blood mononuclear cells using multiple collection and processing methods. PLoS One. (2019) 14:e0225137. doi: 10.1371/journal.pone.0225137, 31809517 PMC6897427

[44] Bustin SA Benes V Garson JA Hellemans J Huggett J Kubista M . The MIQE guidelines: minimum information for publication of quantitative real-time PCR experiments. Clin Chem. (2009) 55:611–22. doi: 10.1373/clinchem.2008.112797, 19246619

[45] Ye J Coulouris G Zaretskaya I Cutcutache I Rozen S Madden TL. Primer-BLAST: a tool to design target-specific primers for polymerase chain reaction. BMC Bioinform. (2012) 13:134. doi: 10.1186/1471-2105-13-134, 22708584 PMC3412702

[46] Radonić A Thulke S Mackay IM Landt O Siegert W Nitsche A. Guideline to reference gene selection for quantitative real-time PCR. Biochem Biophys Res Commun. (2004) 313:856–62. doi: 10.1016/j.bbrc.2003.11.177, 14706621

[47] Livak KJ Schmittgen TD. Analysis of relative gene expression data using real-time quantitative PCR and the 2− ΔΔCT method. Methods. (2001) 25:402–8. doi: 10.1006/meth.2001.1262 11846609

[48] Boom R Sol C Salimans M Jansen C Wertheim-van Dillen P Van der Noordaa J. Rapid and simple method for purification of nucleic acids. J Clin Microbiol. (1990) 28:495–503. doi: 10.1128/jcm.28.3.495-503.1990, 1691208 PMC269651

[49] Al-Shuhaib MBS Hashim HO. Mastering DNA chromatogram analysis in sanger sequencing for reliable clinical analysis. J Genet Eng Biotechnol. (2023) 21:115. doi: 10.1186/s43141-023-00587-6, 37955813 PMC10643650

[50] Kumar S Stecher G Li M Knyaz C Tamura K. MEGA X: molecular evolutionary genetics analysis across computing platforms. Mol Biol Evol. (2018) 35:1547–9. doi: 10.1093/molbev/msy096, 29722887 PMC5967553

[51] Falconer DS Mackay TFC. Introduction to Quantitative Genetics. 4th ed. Harlow, Essex, England: Pearson Prentice Hall (Longman) (1996).

[52] Abramovs N Brass A Tassabehji M. Hardy-Weinberg equilibrium in the large scale genomic sequencing era. Front Genet. (2020) 11:210. doi: 10.3389/fgene.2020.00210, 32231685 PMC7083100

[53] Serbetci I González-Grajales LA Herrera C Ibanescu I Tekin M Melean M . Impact of negative energy balance and postpartum diseases during the transition period on oocyte quality and embryonic development in dairy cows. Front Vet Sci. (2024) 10:1328700. doi: 10.3389/fvets.2023.1328700, 38249554 PMC10797029

[54] Ashar MJ Gonzalez-Rivas PA Dunshea FR Marth CD Chauhan SS. Integrative insights into metabolic, oxidative, and immune adaptations during the transition period in dairy cows: revisiting nutritional strategies and emerging roles of injectable trace minerals. Dairy. (2026) 7:15. doi: 10.3390/dairy7010015

[55] Ameni G Alshamsi SSA Bayissa B Zewude A Mohammed T Degefa BA . Cumulative incidence of ketosis in fresh lactating cows: a case study in the United Arab Emirates. BMC Vet Res. (2025) 21:295. doi: 10.1186/s12917-025-04726-8, 40296118 PMC12036299

[56] Hassan MS Abd-Allah EA El-Amir YO Fayed H Abdelhafeez HH. Histopathological investigation and plasma energy metabolic profile as a diagnostic tools of postpartum metritis and its implication on reproductive performance of dairy cows. EJVS. (2026) 57:253–68. doi: 10.21608/ejvs.2025.352315.2599

[57] Sheldon IM Cronin JG Healey GD Gabler C Heuwieser W Streyl D . Innate immunity and inflammation of the bovine female reproductive tract in health and disease. Reproduction. (2014) 148:R41–51. doi: 10.1530/REP-14-0163, 24890752

[58] Chapwanya A Meade KG Doherty ML Callanan JJ O’Farrelly C. Endometrial immune response in cattle. Reproduction. (2014) 148:41–51.

[59] Gordon S. Phagocytosis: an immunobiologic process. Immunity. (2016) 44:463–75. doi: 10.1016/j.immuni.2016.02.026, 26982354

[60] Sheybani N Bakhtiarizadeh MR Salehi A. An integrated analysis of mRNAs, lncRNAs, and miRNAs based on weighted gene co-expression network analysis involved in bovine endometritis. Sci Rep. (2021) 11:18050. doi: 10.1038/s41598-021-97319-y, 34508138 PMC8433134

[61] O'Neill LA Kishton RJ Rathmell J. A guide to immunometabolism for immunologists. Nat Rev Immunol. (2016) 16:553–65. doi: 10.1038/nri.2016.70, 27396447 PMC5001910

[62] Contreras GA Sordillo LM. Lipid mobilization and inflammatory responses during the transition period of dairy cows. Immunol Microbiol Infect Dis. (2011) 34:281–9. doi: 10.1016/j.cimid.2011.01.004, 21316109

[63] Hotamisligil GS. Inflammation and metabolic disorders. Nature. (2006) 444:860–7. doi: 10.1038/nature05485, 17167474

[64] Sordillo LM Aitken SL. Impact of oxidative stress on the health and immune function of dairy cattle. Vet Immunol Immunopathol. (2009) 128:104–9. doi: 10.1016/j.vetimm.2008.10.305, 19027173

[65] Khatib H Monson RL Schutzkus V Kohl DM Rosa GJ Rutledge JJ. Genetic markers associated with disease resistance in cattle. J Dairy Sci. (2008) 91:784–93. doi: 10.3389/978-2-8325-3096-2 18218766

[66] Al-Sharif M Marghani BH Ateya A. DNA polymorphisms and expression profile of immune and antioxidant genes as biomarkers for reproductive disorders tolerance/susceptibility in Baladi goat. Anim Biotechnol. (2023) 34:2219–30. doi: 10.1080/10495398.2022.2082975, 35671246

[67] Chapwanya A M K Doherty ML Callanan JJ Mee JF O'Farrelly C. Endometrial inflammation and the role of innate immunity in bovine uterine disease. Anim Reprod Sci. (2014) 148:31–7.

[68] Hansen PJ. The immunology of the bovine uterus and implications for reproductive efficiency. J Anim Sci. (2020) 95:skaa088

[69] Kimchi-Sarfaty C Oh JM Kim I-W Sauna ZE Calcagno AM Ambudkar SV . A" silent" polymorphism in the MDR 1 gene changes substrate specificity. Science. (2007) 315:525–8. doi: 10.1126/science.1135308, 17185560

[70] Davies G Genini S Bishop S Giuffra E. An assessment of opportunities to dissect host genetic variation in resistance to infectious diseases in livestock. Animal. (2009) 3:415–36. doi: 10.1017/S1751731108003522, 22444313

[71] Wacka E Nicikowski J Jarmuzek P Zembron-Lacny A. Anemia and its connections to inflammation in older adults: a review. J Clin Med. (2024) 13:2049. doi: 10.3390/jcm13072049, 38610814 PMC11012269

[72] Casaro S Prim J Gonzalez T Bisinotto R Chebel R Marrero M . Unraveling the immune and metabolic changes associated with metritis in dairy cows. J Dairy Sci. (2023) 106:9244–59. doi: 10.3168/jds.2023-23289 37641354

[73] Thrall MA Weiser G Allison RW Campbell TW, (editors). Veterinary hematology and clinical chemistry. 2nd ed. Ames, IA: Wiley-Blackwell (2012).

[74] Baimishev M Baimishev R Eremin S Baimishev H. Hematological parameters that determine the course of labor and the postpartum period in highly productive Holstein cows. Bio Web Conf. (2020) 2:5.

[75] Ramadan MH Mahmoud AEZE Zeidan AE Ahmed AE Hassaneen ASA. Puerperal metritis in crossbreed (Tarentaise X Baladi) cows: metabolism-related biochemical and haematological changes. J Adv Vet Res. (2020) 10:96–104.

[76] Perumal P De A Sunder J Bhattacharya D. Physiological, heamatological, biochemical and endocrinological profiles changes in crossbred cows affected with metritis under tropical island ecosystem. Int J Bio-Resour Stress Manage. (2022) 13:155–161.

[77] Jayaganthan P Puvarajan B Satheshkumar S Krishnakumar K Ravikumar K Raja S . Hematobiochemical alterations and antimicrobial resistance profiles of uterine pathogens in postpartum metritic crossbred cows. J Andaman Sci Assoc. (2025) 30:80–92.

[78] Torres E Mellado M Leyva C García JE Véliz FG Hernández-Bustamante J. Serum metabolites and body condition score associated with metritis, endometritis, ketosis, and mastitis in Holstein cows. Pesq Agrop Brasileira. (2020) 55:e01308. doi: 10.1590/s1678-3921.pab2020.v55.01308

[79] Jan M Kumar H Kumar S Malla W Sharma R. Comparative biochemical profiles, utero-ovarian function, and fertility of the postpartum buffalo with and without subclinical endometritis. Trop Anim Health Prod. (2021) 53:73. doi: 10.1007/s11250-020-02502-4, 33400003

[80] Kaufmann TB Drillich M Tenhagen B-A Heuwieser W. Correlations between periparturient serum concentrations of non-esterified fatty acids, beta-hydroxybutyric acid, bilirubin, and urea and the occurrence of clinical and subclinical postpartum bovine endometritis. BMC Vet Res. (2010) 6:47. doi: 10.1186/1746-6148-6-47, 20979598 PMC2988005

[81] Abuelo A Mann S Contreras GA. Metabolic factors at the crossroads of periparturient immunity and inflammation. Vet Clin North Am Food Anim Pract. (2023) 39:203–18. doi: 10.1016/j.cvfa.2023.02.012, 37032303

[82] de Souza Pereira FN. Exploring the Impacts of Clinical Resolution of Metritis on Dairy Cows Through an Epidemiological and a Molecular Lens. Pullman, WA (Washington), USA: Washington State University (2025).

[83] Pascottini OB LeBlanc SJ Gnemi G Leroy JL Opsomer G. Genesis of clinical and subclinical endometritis in dairy cows. Reproduction. (2023) 166:R15–24. doi: 10.1530/REP-22-0452, 37294111

[84] Qiao K Jiang R Contreras GA Xie L Pascottini OB Opsomer G . The complex interplay of insulin resistance and metabolic inflammation in transition dairy cows. Animals. (2024) 14:832. doi: 10.3390/ani14060832, 38539930 PMC10967290

[85] Bruinjé T Pascottini OB LeBlanc S. Inflammatory and metabolic markers in postpartum dairy cows developing chronic reproductive tract disease: a case-control study. J Dairy Sci. (2026) 109:572–83. doi: 10.3168/jds.2025-26910 41130400

[86] Ohtsuka H. Watanabe C. Kohiruimaki M. Ando T. Watanabe D. Masui M. Comparison of two different nutritive conditions against the changes in peripheral blood mononuclear cells of periparturient dairy cows. J Vet Med Sci (2006) 68: p. 1161–1166. Doi: doi: 10.1292/jvms.68.1161, 17146172 17146172

[87] Beckett LM Kendall SJ Casey TM Donkin SS White HM. Invited review: fueling milk production carbon by carbon: regulation of hepatic glucose production in dairy cattle. J Dairy Sci. (2025) 108:11787–801. doi: 10.3168/jds.2025-26987, 40945784

[88] Cui L Wang H Ding Y Li J Li J. Changes in the blood routine, biochemical indexes and the pro-inflammatory cytokine expressions of peripheral leukocytes in postpartum dairy cows with metritis. BMC Vet Res. (2019) 15:157. doi: 10.1186/s12917-019-1912-y, 31113485 PMC6528309

[89] Ruiz-García L Arévalo I Carcelén F Pizarro J Sandoval-Monzón R. Association between serum calcium levels and the presentation of postpartum endometritis in housed dairy cows. Res Vet Sci. (2022) 144:92–7. doi: 10.1016/j.rvsc.2022.01.015, 35093721

[90] Chandler T Westhoff T LaPierre P Frizzarini W Hernandez L Overton T . Eucalcemia during lipopolysaccharide challenge in postpartum dairy cows: II. Calcium dynamics. J Dairy Sci. (2023) 106:3601–14. doi: 10.3168/jds.2022-22775 37002137

[91] Arechiga-Flores CF Cortés-Vidauri Z Hernández-Briano P Lozano-Domínguez RR López-Carlos MA Macías-Cruz U . Hypocalcemia in the dairy cow. Review. Rev Mex Cienc Pecu. (2022) 13:1025–54. doi: 10.22319/rmcp.v13i4.5277

[92] Bruinjé TC LeBlanc SJ. Invited review: inflammation and health in the transition period influence reproductive function in dairy cows. Animals. (2025) 15:633. doi: 10.3390/ani15050633, 40075916 PMC11898178

[93] Perumal P Chaurasia D De A Bhattacharya D Sunder J Bhowmick S . Effect of clinical endometritis on physiological, hematological, biochemical and endocrinological profiles in crossbred cows under tropical island ecosystem. Indian J Anim Sci. (2020) 90:1296–9. doi: 10.56093/ijans.v90i9.109493

[94] Trevisi E Cattaneo L Piccioli-Cappelli F Mezzetti M Minuti A. International symposium on ruminant physiology: the immunometabolism of transition dairy cows from dry-off to early lactation—lights and shadows. J Dairy Sci. (2025) 108:7662–74. doi: 10.3168/jds.2024-25790 39778800

[95] Abdoon ASS Soliman SS Nagy AM. Uterotubal junction of the bovine (*Bos taurus*) versus the dromedary camel (*Camelus dromedarius*): histology and histomorphometry. Reprod Domest Anim. (2024) 59:e14665. doi: 10.1111/rda.14665, 38973694

[96] Karsch FJ Battaglia DF Breen KM Debus N Harris TG. Mechanisms for ovarian cycle disruption by immune/inflammatory stress. Stress. (2002) 5:101–12. doi: 10.1080/10253890290027868, 12186688

[97] Cheong S H Filho O G S Absalón-Medina V A Pelton S H Butler W R Gilbert R O. Metabolic and endocrine differences between dairy cows that do or do not ovulate first postpartum dominant follicles. Biol Reprod (2016) 94(1): p. 18, –11. Doi: doi: 10.1095/biolreprod.114.127076. 26632612

[98] Soliman SS. Ovarian morphometry and follicular population in non-pregnant and pregnant *camelus dromedaries*. EJVS. (2025) 56:1931–7. doi: 10.21608/ejvs.2025.342608.2545

[99] Williams EJ Fischer DP Noakes DE England GC Rycroft A Dobson H . The relationship between uterine pathogen growth density and ovarian function in the postpartum dairy cow. Theriogenology. (2007) 68:549–59. doi: 10.1016/j.theriogenology.2007.04.056, 17574659 PMC2702080

[100] Abdoon ASS Soliman SS Hussein NS Haggag SH El-Sanea AM Abdel-Hamid A-HZ. Metabolomic profile of dromedary camel follicular fluid during the breeding and non-breeding seasons. Sci Rep. (2025) 15:8923. doi: 10.1038/s41598-025-91710-9, 40087336 PMC11909251

[101] El-Deeb W Abdelghani M A Alhaider A Al-Hammadi M Gomaa N Venugopala K Exploring oxidative stress, immunological and metabolic biomarkers in dairy cows with postpartum pyometra. Reprod Domest Anim (2024) 59:e14559. doi: DOI: 10.1111/rda.14559, 38591742. 38591742

[102] Serhiienko V Koshevoy V Naumenko S Kotyk B Ilina O Shchepetilnikov Y . Oxidative stress markers, antioxidant balance, and protein metabolism in dogs with acute prostatitis. World Vet J. (2025) 15:167–75. doi: 10.54203/scil.2025.wvj19

[103] Sheldon IM Molinari PC Ormsby TJ Bromfield JJ. Preventing postpartum uterine disease in dairy cattle depends on avoiding, tolerating and resisting pathogenic bacteria. Theriogenology. (2020) 150:158–65. doi: 10.1016/j.theriogenology.2020.01.017, 31973964 PMC7234917

[104] Skliarov P Fedorenko S Naumenko S Bilyi D Koshevoy V Petrusha V . Cows postpartum polymorbid pathology. J Adv Vet Res. (2023) 13:1730–6.

[105] Shaukat A Hanif S Shaukat I Rajput SA Shukat R Huang S-c . Up-regulation of inflammatory, oxidative stress, and apoptotic mediators via inflammatory, oxidative stress, and apoptosis-associated pathways in bovine endometritis. Microb Pathog. (2024) 191:106660. doi: 10.1016/j.micpath.2024.106660, 38657710

[106] Liu X Zhang W Wang Y Gao Q. Activation of TLR4/NF-κB signaling in bovine endometrial epithelial cells induced by *Escherichia coli* LPS. Front Immunol. (2023) 14:1193745

[107] Biswal P Lathwal S Baithalu RK Nag P Kumar S. Total antioxidant capacity, neutrophil profile, *in vitro* phagocytic activity, myeloperoxidase (MPO) activity and IL-8 status in uterine infected Murrah buffaloes during peripartum period. Indian J Anim Sci. (2022) 92:32–7. doi: 10.56093/ijans.v92i1.120914

[108] Niroomandi E Maleki S Abdollahpour G Zakian A Ahmadvand H. The effect of natural infection with different *Leptospira interrogans* serovars on oxidative stress biomarkers and acute-phase responses in horses and cattle. Vet Clin Pathol. (2022) 51:84–92. doi: 10.1111/vcp.13042, 35179227

[109] Lee JS Kim C. Role of CARD9 in cell-and organ-specific immune responses in various infections. Int J Mol Sci. (2024) 25:2598. doi: 10.3390/ijms25052598, 38473845 PMC10932402

[110] Wu R Kang R Tang D. Mitochondrial ACOD1/IRG1 in infection and sterile inflammation. J Intensive Med. (2022) 2:78–88. doi: 10.1016/j.jointm.2022.01.001, 36789185 PMC9924012

