# Supplementary material for: Identification of reproductive and molecular biomarkers associated with clinical metritis in dromedary camels (Camelus dromedarius)
Source: Front Vet Sci. 2026 Jul 22;13:1890020. doi: 10.3389/fvets.2026.1890020 (PMC13437421; doi:10.3389/fvets.2026.1890020)
Supplement: Supplementary file 1 [file Data_Sheet_1.pdf]

```
XM_031450839.2      TACCTGCGGCAGTGCAAGGTCCTGAACCCTGATGATGAGGAGCAGGTGCTCAGCGACCCC   60
H                   TACCTGCGGCAGTGCAAGGTCCTGAACCCTGATGATGAGGAGCAGGTGCTCAGCGACCCC   60
M                   TACCTGCGGCAGTGCAAGGTCCTGAACCCTGATGATGAGGAGCAGGTGCTCAGCGACCCC   60
                    ************************************************************

XM_031450839.2      AACCTGGTCATCCGCAAGCGGAAAGTGGGTGTGCTCCTGGACATCCTGCAGCGGACTGGC   120
H                   AACCTGGTCATCCGCAAGCGGAAAGTGGGTGTGCTCCTGGACATCCTGCAGCGGACTGGC   120
M                   AACCTGGTCATCCGCAAGCGGAAAGTGGGTGTGCTCCTGGACATCCTGCAGCGGACTGGC   120
                    ************************************************************

XM_031450839.2      CACAAGGGCTATGTGGCCTTCCTTGAGAGCCTGGAGCTGTACTACCCACAGCTCTACAAG   180
H                   CACAAGGGCTACGTGGCCTTCCTTGAGAGCCTGGAGCTGTACTACCCACAGCTCTACAAG   180
M                   CACAAGGGCTATGTGGCCTTCCTTGAGAGCCTGGAGCTGTACTACCCACAGCTCTACAAG   180
                    *********** ************************************************

XM_031450839.2      AAGGTCACAGGCAAGGAGCCCACCCGCGTCTTCTCCATGATCATCGATGCGTCCGGGGAG   240
H                   AAGGTCACAGGCAAGGAGCCCACCCGCGTCTTCTCCATGATCATCGATGCGTCCGGGGAG   240
M                   AAGGTCACAGGCAAGGAGCCCACCCGCGTCTTCTCCATGATCATCGATGCGTCCGGGGAG   240
                    ************************************************************

XM_031450839.2      TCGGGCCTGATGCAGCTGCTGATGAGCGAGGTGATGAAGCTGCAGAAGAAGGTGCAGGAC   300
H                   TCGGGCCTGATGCAGCTGCTGATGAGCGAGGTGATGAAGCTGCAGAAGAAGGTGCAGGAC   300
M                   TCGGGCCTGACGCAGCTGCTGATGAGCGAGGTGATGAAGCTGCAGAAGAAGGTGCAGGAC   300
                    ********** *************************************************

XM_031450839.2      CTGACGGTGCTGCTGAGCTCCAAGGACGACCTGATCAAGGAGCTGCGGGTGAAGGACAGC   360
H                   CTGACGGTGCTGCTGAGCTCCAAGGACGACCTGATCAAGGAGCTGCGGGTGAAGGACAGC   360
M                   CTGACGGTGCTGCTGAGCTCCAAGGACGACCTGATCAAGGAGCTGCGGGTGAAGGACAGC   360
                    ************************************************************

XM_031450839.2      CTGCTGCGCAAGCACCAGGAGCGCGTGCAGCGGCTCAAGGAGGCTTGCGAGGC 413
H                   CTGCTGCGCAAGCACCAGGAGCGCGTGCAGCGGCTCAAGGAGGCTTGCGAGGC 413
M                   CTGCTGCGCAAGCACCAGGAGCGCGTGCAGCGGCTCAAGGAGGCTTGCGAGGC 413
                    *****************************************************
```

Figure S1: Comparative alignment of coding DNA sequence (CDS) fragments of the *CARD9* gene (413-bp) between healthy and metritis she-camel relative to the corresponding reference sequence retrieved from GenBank (XM_031450839.2). H= healthy; and M= Metritis. Asterisks represent similarity.

```
XM_010988253.3      CTGCGCTGCCAGCTCTCGGTGGCCAACCACAGGACTCAGGGCGAGATTCATCTCCAAGTG   60
H                   CTGCGCTGCCAGCTCTCGGTGGCCAACCACAGGACTCAGGGCGAGATTCATCTCCAAGTG   60
M                   CTGCGCTGCCAGCTCTCGGTGGCCAACCACAGGACTCAGGGCGAGATTCATCTCCAAGTG   60
                    ************************************************************

XM_010988253.3      GAGTATGCCCCCAAGGGTGTGGAGATCCTCCTCAGCCCTTCAGGGAGGAACATCCTTCCA   120
H                   GAGTATGCCCCCAAGGGTGTGGAGATCCTCCTCAGCCCTTCAGGGAGGAACATCCTTCCA   120
M                   GAGTATGCCCCCAAGGGTGTGGAGATCCTCCTCAGCCCTTCAGGGAGGAACATCCTTCCA   120
                    ************************************************************

XM_010988253.3      GGTGATCTGGTCACACTCACCTGCCAGGTGAACAGCAGTCACCCTCAGGTCAGTTCCGTG   180
H                   GGTGATCTGGTCACACTCACCTGCCAGGTGAACAGCAGTCACCCTCAGGTCAGTTCCGTG   180
M                   GGTGATCTGGTCACACTCACCTGCCAGGTGAACAGCAGTCACCCTCAGGTCAGTTCCGTG   180
                    ************************************************************

XM_010988253.3      AAGTGGGTCAAGGATGGGACACACCTCAAAGACCAGAGTCGTGTCCTGCAGTTGCCCCGG   240
H                   AAGTGGGTCAAGGATGGGACACACCTCAAAGACCAGAGTCGTGTCCTGCAGTTGCCCCGG   240
M                   CAGTGGGTCAAGGATGGGACACACCTCAAAGACCAGAGTCGTGTCCTGCAGTTGCCCCGG   240
                     ***********************************************************

XM_010988253.3      GCAGCCTGGGATGACGCTGGCGTCTACACCTGCCTCGCTGGGAATGGCGTGGGCTCTTCG   300
H                   GCAGCCTGGGATGACGCTGGCGTCTACACCTGCCTCGCTGGGAATGGCGTGGGCTCTTCG   300
M                   GCAGCCTGGGATGACGCTGGCGTCTACACCTGCCTCGCTGGGAATGGCGTGGGCTCTTCG   300
                    ************************************************************

XM_010988253.3      GTCTCACCACCGGTCAGCCTCCACATCTTCATGGCTGAG      339
H                   GTCTCACCACCGGTCAGCCTCCACATCTTCATGGCTGAG      339
M                   GTCTCACCACCGGTCAGCCTCCACATCTTCATGGCTGAG      339
                    ***************************************
```

Figure S2: Comparative alignment of coding DNA sequence (CDS) fragments of the *SIGLEC1* gene (339-bp) between healthy and metritis she-camel relative to the corresponding reference sequence retrieved from GenBank (XM_010988253.3). H= healthy; and M= Metritis. Asterisks represent similarity.

```
XM_010981998.3      CACTACACGTGTGAAGTCACTTGGCAGACCTCTGATGGCAACAAAGTCATGAGAGATAAG   60
H                   CACTACACGTGTGAAGTCACTTGGCAGACCTCTGATGGCAACAAAGTCATGAGAGATAAG   60
M                   CACTACACGTGTGAAGTCACTTGGCAGACCTCTGATGGCAACAAAGTCATGAGAGATAAG   60
                    ************************************************************

XM_010981998.3      ATCATTGAACTCCGTGTCCAGAAACACTTCTCAAAGCCACTTGAGATCAAGACTAAGGCA   120
H                   ATCATTGAACTCCGTGTCCAGAAACACTTCTCAAAGCCACTTGAGATCAAGACTAAGGCA   120
M                   ATCATTGAACTCCGTGTCCAGAAACACTTCTCAAAGCCACTTGAGATCAAGACTAAGGCA   120
                    ************************************************************

XM_010981998.3      CCCACAACTATGCAATCCCCCGTGGAAGCAACATCCAGAGTGAACTCATCTTGGGGCTGG   180
H                   CCCACAACTATGCAATCCCCCGTGGAAGCAACATCCAGAGTGAACTCATCTTGGGGCTGG   180
M                   CCCACAACTATGCAATCCCCCGTGGAAGCAACATCCAGAGTGAGCTCATCTTGGGGCTGG   180
                    ******************************************* ****************

XM_010981998.3      ACCACTAAAGCTGATAACTACCTTGAGAAGACAAGTGCTGGGCCAGGAAAGGGACTACCT   240
H                   ACCACTAAAGCTGATAACTACCTTGAGAAGACAAGTGCTGGGCCAGGAAAGGGACTACCT   240
M                   ACCACTAAAGCTGATAACTACCTTGAGAAGACAAGTGCTGGGCCAGGAAAGGGACTACCT   240
                    ************************************************************

XM_010981998.3      ATCTTTGCCATCATTCTCATCATCTCCTTGTGCTGTTTAGTTGTAATCACCATGGCTTAT   300
H                   ATCTTTGCCATCATTCTCATCATCTCCTTGTGCTGTTTAGTTGTAATCACCATGGCTTAT   300
M                   ATCTTTGCCATCATTCTCATCATCTCCTTGTGCTGTTTAGTTGTAATCACCATGGCTTAT   300
                    ************************************************************

XM_010981998.3      ATCATTCTCTGCCGGAAGACATCTCAACAGGATGTCTATGAAGTTACCAGGGTGCATGCC   360
H                   ATCATTCTCTGCCGGAAGACATCTCAACAGGATGTCTATGAAGTTACCAGGGTGCATGCC   360
M                   ATCATTCTCTGCCGGAAGACATCTCAACAGGATGTCTATGAAGTTACCAGGGTGCATGCC   360
                    ************************************************************

XM_010981998.3      AGAGAGGCCAGTGACTCTGGTGAAACCATGAGGGTGGCCATCTTCGTGAGTGGTTGCTCC   420
H                   AGAGAGGCCAGTGACTCTGGTGAAACCATGAGGGTGGCCATCTTCATGAGTGGTTGCTCC   420
M                   AGAGAGGCCAGTGACTCTGGTGAAACCATGAGGGTGGCCATCTTCGTGAGTGGTTGCTCC   420
                    ********************************************* **************

XM_010981998.3      AGTGAAGAGCCAGCTTCCCAGACTCTGGGCAACAATTACTCTGATGAG        468
H                   AGTGAAGAGCCAGCTTCCCAGACTCTGGGCAACAATTACTCTGATGAG        468
M                   AGTGAAGAGCCAGCTTCCCAGACTCTGGGCAACAATTACTCTGATGAG        468
                    ************************************************
```

Figure S3: Comparative alignment of coding DNA sequence (CDS) fragments of the *VSIG4* gene (468-bp) between healthy and metritis she-camel relative to the corresponding reference sequence retrieved from GenBank (XM_010981998.3). H= healthy; and M= Metritis. Asterisks represent similarity.

```
XM_031461630.2      ATGGACCCGGCCGAGGCGGTACTGCAGGAAAAAGCGCTCAAGTTTATGACTTACAACAGC    60
H                   ATGGACCCGGCCGAGGCGGTACTGCAGGAAAAAGCGCTCAAGTTTATGACATACAACAGC    60
M                   ATGGACCCGGCCGAGGCGGTACTGCAGGAAAAAGCGCTCAAGTTTATGACTTACAACAGC    60
                    *************************************************** ********

XM_031461630.2      TGTGCCAGACTCTGCTTAAATCAAGAGACAGTATGTCTAGCAAGCACTGCTATGAAGACT    120
H                   TGTGCCAGACTCTGCTTAAATCAAGAGACAGTATGTCTAGCAAGCACTGCTATGAAGACT    120
M                   TGTGCCAGACTCTGCTTAAATCAAGAGACAGTATGTCTAGCAAGCACTGCTATGAAGACT    120
                    ************************************************************

XM_031461630.2      GAAAATTGTGTGGCCAAAACAAAACTTGCTAATGGCACTTCCAGTATGATTGTGCCCAAG    180
H                   GAAAATTGTGTGGCCAAAACAAAACTTGCTAATGGCACTTCCAGTATGATTGTGCCCAAG    180
M                   GAAAATTGTGTGGCCAAAACAAAACTTGCTAATGGCACTTCCAGTATGATTGTGCCCAAG    180
                    ************************************************************

XM_031461630.2      CAACGGAAACTCTCGGCAAGCTATGAGAAGGAAAAGGAACTGTGTGTCAAATATTTTGAG    240
H                   CAACGGAAACTCTCGGCAAGCTATGAGAAGGAAAAGGAACTGTGTGTCAAATATTTTGAG    240
M                   CAACGGAAACTCTCAGCAAGCTATGAGAAGGAAAAGGAACTGTGTGTCAAATATTTTGAG    240
                    ************** *********************************************

XM_031461630.2      CAGTGGTCAGAGTCTGATCAAGTGGAATTTGTGGAACATCTTATATCCCAAATGTGTCAT    300
H                   CAGTGGTCAGAGTCTGATCAAGTGGAATTTGTGGAACATCTTATATCCCAAATGTGTCAT    300
M                   CAGTGGTCAGAGTCTGATCAAGTGGAATTTGTGGAACATCTTATATCCCAAATGTGTCAT    300
                    ************************************************************

XM_031461630.2      TACCAACATGGGCACATAAACTCCTATCTTAAACCTATGTTGCAGAGGGATTTCATAACT    360
H                   TACCAACATGGGCACATAAACTCCTATCTTAAACCTATGTTGCAGAGGGATTTCATAACT    360
M                   TACCAACATGGGCACATAAACTCCTATCTTAAACCTATGTTGCAGAGGGATTTCATAACT    360
                    ************************************************************

XM_031461630.2      GCTCTGCCAGCTCGAGGTTTGGATCATATTGCTGAGAACATTCTGTCATACCTGGATGCC    420
H                   GCTCTGCCAGCTCGAGGTTTGGATCATATTGCTGAGAACATTCTGTCATACCTGGATGCC    420
M                   GCTCTGCCAGCTCGAGGTTTGGATCATATTGCTGAGAACATTCTGTCATACCTGGATGCC    420
                    ************************************************************

XM_031461630.2      AAATCACTATGTGCTGCTGAACTTGTGTGCAAGGAATGGTACCGAGTGACATCT          474
H                   AGATCACTATGTGCTGCTGAACTTGTGTGCAAGGAATGGTACCGAGTGACATCT          474
M                   AAATCACTATGTGCTGCTGAACTTGTGTGCAAGGAATGGTACCGAGTGACATCT          474
                    * ****************************************************
```

Figure S4: Comparative alignment of coding DNA sequence (CDS) fragments of the *BTRC* gene (474-bp) between healthy and metritis she-camel relative to the corresponding reference sequence retrieved from GenBank (XM_031461630.2). H= healthy; and M= Metritis. Asterisks represent similarity.

```
XM_031444155.2      AGGTGGCGCAATTCAATGCTAATCGTCAACCTGTTGGCCATTGCTGGTGGCTGCCTGATG   60
H                   AGGTGGCGCAATTCAATGCTAATCGTCAACCTGTTGGCCATTGCTGGTGGCTGCCTGATG   60
M                   AGGTGGCGCAATTCAATGCTAATCGTCAACCTGTTGGCCATTGCTGGTGGCTGCCTGATG   60
                    ************************************************************

XM_031444155.2      GGTTTCTGCAAAATAGCGGAGTCGGTTGAAATGCTGATCCTGGGCCGACTGATTATTGGC   120
H                   GGCTTCTGCAAAATAGCGGAGTCGGTTGAAATGCTGATCCTGGGCCGACTGATTATTGGC   120
M                   GGTTTCTGCAAAATAGCGGAGTCGGTTGAAATGCTGATCCTGGGCCGACTGATTATTGGC   120
                    ** *********************************************************

XM_031444155.2      ATCTTCTGTGGACTCTGCACAGGATTTGTCCCCATGTACATTGGGGAGATATCTCCTACT   180
H                   ATCTTCTGTGGACTCTGCACAGGATTTGTCCCCATGTACATTGGGGAGATATCTCCTACT   180
M                   ATCTTCTGTGGACTCTGCACAGGATTTGTCCCCATGTACATTGGGGAGATATCTCCTACT   180
                    ************************************************************

XM_031444155.2      GCCCTGCGGGGGGCCTTCGGCACTCTCAACCAGCTGGGCATCGTTATCGGGATTCTGGTG   240
H                   GCCCTGCGGGGGGCCTTCGGCACTCTCAACCAGCTGGGCATCGTTATCGGGATTCTGGTG   240
M                   GCCCTGCGGGGGGCCTTCGGCACTCTCAACCAGCTGGGCATCGTTATCGGGATTCTGGTG   240
                    ************************************************************

XM_031444155.2      GCCCAGATCTTTGGTCTGAAAGTCATCATGGGGACTGAAGAGCTCTGGCCACTGCTCTTG   300
H                   GCCCAGATCTTTGGTCTGAAAGTCATCATGGGGACTGAAGAGCTCTGGCCACTGCTCTTG   300
M                   GCCCAGATCTTTGGTCTGAAAGTCATCATGGGGACTGAAGAGCTCTGGCCACTGCTCTTG   300
                    ************************************************************

XM_031444155.2      GGCTTCACCATTATTCCAGCTGTCCTGCAATGTGCTGCGCTGCCATTTTGCCCCGAAAGT   360
H                   GGCTTCACCATTATTCCAGCTGTCCTGCAATGTGCTGCGCTGCCATTTTGCCCCGAAAGT   360
M                   GGCTTCACCATTATTCCAGCTGTCCTGCAATGTGCTGCGCTGCCATTTTGCCCCGAAAGT   360
                    ************************************************************

XM_031444155.2      CCGAGATTCTTGCTCATTAACAGA   384
H                   CCGAGATTCTTGCTCATTAACAGA   384
M                   CCGAGATTCTTGCTCATTAACAGA   384
                    ************************
```

Figure S5: Comparative alignment of coding DNA sequence (CDS) fragments of the *SLC2A3* gene (384-bp) between healthy and metritis she-camel relative to the corresponding reference sequence retrieved from GenBank (XM_031444155.2). H= healthy; and M= Metritis. Asterisks represent similarity.

```
XM_010996663.3      ACGCTGCTTGATGGTGCCATCGCTGTCCCATCGTTCCACAAATGCCAGATCAACAGGCCA   60
H                   ACGCTGCTTGATGGTGCCATCGCTGTCCCATCGTTCCACAAATGCCAGATCAACAGGCCA   60
M                   ACGCTGCTTGATGGTGCCATCGCTGTCCCATCGTTCCACAAATGCCAGATCAACAGGCCA   60
                    ************************************************************

XM_010996663.3      CAGGTGAGAGAGCTGCTGGGTAAGGTGGAGCTGGAGCACCCTCGAGACAACCTCCCAAAC   120
H                   CAGGTGAGAGAGCTGCTGGGTAAGGTGGAGCTGGAGCACCCTCGAGACAACCTCCCAAAC   120
M                   CAGGTGAGAGAGCTGCTGGGTAAGGTGGAGCTGGAGCACCCTCGAGACAACCTCCCAAAC   120
                    ************************************************************

XM_010996663.3      TTCAACACACTGTACTGCGAGGTAAGTGTCGTCCTCAAGGATGGAGCCATCTTCACAGAG   180
H                   TTCAACACACTGTACTGCGAGGTAAGTGTCGTCCTCAAGGATGGAGCCATCTTCACAGAG   180
M                   TTCAACACACTGTACTGCGAGGTAAGTGTCGTCCTCAAGGATGGAGCCATCTTCACAGAG   180
                    ************************************************************

XM_010996663.3      CGCTGTGATACCTTCTACGGTCACTGGAGGAAGCCTCTAAGCCAGAAGGACCTACGGGAA   240
H                   CGCTGTGATACCTTCTACGGTCACTGGAGGAAGCCTCTAAGCCAGAAGGACCTACTGGAA   240
M                   CGCTGTGATACCTTCTACGGTCACTGGAGGAAGCCTCTAAGCCAGAAGGACCTACGGGAA   240
                    ******************************************************* ****

XM_010996663.3      AAGTTCAGAGCCAATGCCTCCAGGGTGCTGTCCTGCCACACAGTAGAAAGGCTTATAGAG   300
H                   AAGTTCAGAGCCAATGCCTCCAGGGTGCTGTCCTGCCACACAGTAGAAAGGCTTATAGAG   300
M                   AAGTTCAGAGCCAATGCCTCCAGGGTGCTGTCCTGCCACACAGTAGAAAGGCTTATAGAG   300
                    ************************************************************

XM_010996663.3      CTAGTAGAAAACCTAGAAGTCGTAGGAGACTGTTCTGTGCTGACTGCTCTTCTGAAAGAA   360
H                   CTAGTAGAAAACCTAGAAGTCGTAGGAGACTGTTCTGTGCTGACTGCTCTTCTGAAAGAA   360
M                   CTAGTAGAAAACCTAGAAGTCGTAGGAGACTGTTCTGTGCTGACTGCTCTTCTGAAAGAA   360
                    ************************************************************

XM_010996663.3      CTCTCTCCACCAGAAGTGGCTTC   383
H                   CTCTCTCCACCAGAAGTGGCTTC   383
M                   CTCTCTCCACCAGAAGTGGCTTC   383
                    ***********************
```

Figure S6: Comparative alignment of coding DNA sequence (CDS) fragments of the *ACOD1* gene (383-bp) between healthy and metritis she-camel relative to the corresponding reference sequence retrieved from GenBank (XM_010996663.3). H= healthy; and M= Metritis. Asterisks represent similarity.

```
XM_031448592.2      ACAATTCCGCTCTGCTCTGCCCAGTGGGAGCGGATGTTCAATACGTCCCGGATCCCAGGA   60
H                   ACAATTCCGCTCTGCTCTGCCCAGTGGGAGCGGATGTTCAATACGTCCCGGATCCCAGGA   60
M                   ACAATTCCGCTCTGCTCTGCCCAGTGGGAGCGGATGTTCAATACGTCCCGGATCCCAGGA   60
                    ************************************************************

XM_031448592.2      GAGGAAACAGACACCATCCAGCACATGCGGGACAGCAAGCACATAGTTGTGTTCCACAGA   120
H                   GAGGAAACAGACACCATCCAGCACATGCGGGACAGCAAGCACATAGTTGTGTTCCACAGA   120
M                   GAGGAAACAGACACCATCCAGCACATGCGGGACAGCAAGCACATAGTCGTGTTCCACAGA   120
                    *********************************************** ************

XM_031448592.2      GGGCGCTACTTCAAGGTCTGGCTCTACCACGACGGCAGGCTGTTGAAGCCCCGAGAAATC   180
H                   GGGCGCTACTTCAAGGTCTGGCTCTACCACGACGGCAGGCTGTTGAAGCCCCGAGAAATC   180
M                   GGGCGCTACTTCAAGGTCTGGCTCTACCACGACGGCAGGCTGTTGAAGCCCCGAGAAATC   180
                    ************************************************************

XM_031448592.2      GAACAGCAGATGCAGAGGATTTTGGACGACCCGTCCGAGCCCCAGGCTGGGGAGGAGAAG   240
H                   GAACAGCAGATGCAGAGGATTTTGGATGACCCGTCCGAGCCCCAGGCTGGGGAGGAGAAG   240
M                   GAACAGCAGATGCAGAGGATTTTGGACGACCCGTCCGAGCCCCAGGCTGGGGAGGAGAAG   240
                    ************************* **********************************

XM_031448592.2      CTGGCGGCCCTCACTGCGGGAGAGAGAGTACCCTGGGCCAGGTGTCGCCAAGCCTATTTT   300
H                   CTGGCGGCCCTCACTGCGGGAGAGAGAGTACCCTGGGCCAGGTGTCGCCAAGCCTATTTT   300
M                   CTGGCAGCCCTCACTGCGGGAGAGAGAGTACCCTGGGCCAGGTGTCGCCAAGCCTATTTT   300
                    ***** ******************************************************

XM_031448592.2      GGACAGGGCAAAAATAAGCAGTCCCTTGATGCCGTGGAGAAAGCAGCGTTCTTCGTGACG   360
H                   GGACAGGGCAAAAATAAGCAGTCCCTTGATGCCGTGGAGAAAGCAGCGTTCTTCGTGACG   360
M                   GGACAGGGCAAAAATAAGCAGTCCCTTGATGCCGTGGAGAAAGCAGCGTTCTTCGTGACG   360
                    ************************************************************

XM_031448592.2      TTGGACGAAACCGAACAAGGATACAGGAAGGAAGACGTGGA   401
H                   TTGGACGAAACCGAACAAGGATACAGGAAGGAAGACGTGGA   401
M                   TTGGACGAAACCGAACAAGGATACAGGAAGGAAGACGTGGA   401
                    *****************************************
```

Figure S7: Comparative alignment of coding DNA sequence (CDS) fragments of the *CPT1A* gene (401-bp) between healthy and metritis she-camel relative to the corresponding reference sequence retrieved from GenBank (XM_031448592.2). H= healthy; and M= Metritis. Asterisks represent similarity.

```
XM_064479318.1      GTCCGAAAGCAGTGTGCACTAGCTGCCCTGAGAGACGTCAAAAGTTACCTGACGAAGGAA    60
H                   GTCCGAAAGCAGTGTGCACTAGCTGCCCTGAGAGACGTCAAAAGTTACCTGACGAAGGAA    60
M                   GTCCGAAAGCAGTGTGCACTAGCTGCCCTGAGAGACGTCAAAAGTTACCTGACGAAGGAA    60
                    ************************************************************

XM_064479318.1      AGGGGCCAGATTGCAGTTTTCGATGCTACCAATACTACTAGAGAGAGGAGACATATGATC    120
H                   AGGGGCCAGATTGCAGTTTTCGATGCTACCAATACTACTAGAGAGAGGAGACATATGATC    120
M                   AGGGGCCAGATTGCAGTTTTCGATGCTACCAATACTACTAGAGAGAGGAGACATATGATC    120
                    ************************************************************

XM_064479318.1      CTTCATTTTGCCAAAGAAAATGATTTCAAGGTGTTTTTCATCGAGTCTGTGTGCGATGAC    180
H                   CTTCATTTTGCCAAAGAAAATGATTTCAAGGTGTTTTTCATCGAGTCTGTGTGCGATGAC    180
M                   CTTCATTTTGCCAAAGAAAATGCTTTCAAGGTGTTTTTCATCGAGTCTGTGTGCGATGAC    180
                    ********************** *************************************

XM_064479318.1      CCCACGGTTGTAGCCTCAAACATCATGGAAGTGAAAATCTCCAGCCCGGATTACAAAGAT    240
H                   CCCACGGTTGTAGCCTCAAACATCATGGAAGTGAAAATCTCCAGCCCGGATTACAAAGAT    240
M                   CCCACGGTTGTAGCCTCAAACATCATGGAAGTGAAAATCTCCAGCCCGGATTACAAAGAT    240
                    ************************************************************

XM_064479318.1      TGCAACTCAGCAGAAGCCATGGACGACTTCATGAAGAGAATTAATTGCTATGAAGCCAGC    300
H                   TGCAACTCAGCAGAAGCCATGGACGACTTCATGAAGAGAATTAATTGCTATGAAGCCAGC    300
M                   TGCAACTCAGCAGAAGCCATGGACGACTTCATGAAGAGAATTAATTGCTATGAAGCCAGC    300
                    ************************************************************

XM_064479318.1      TACCAGCCCCTCGACCCCGATAAATGTGACAGGGACCTGTCCCTGATCAAGGTGATCGAC    360
H                   TACCAGCCCCTCGACCCCGATAAATGTGACAGGGACCTGTCCCTGATCAAGGTGATCGAC    360
M                   TACCAGCCCCTCGACCCCGATAAATGTGACAGGGACCTGTCCCTGATCAAGGTGATCGAC    360
                    ************************************************************

XM_064479318.1      GTGGGCCGGCGGTTCCTGGTGAACAGGGTTCAGGACCACATCCAGAGCCGGATCGTCTAC    420
H                   GTGGGCCGGCGGTTCCTGGTGAACAGGGTTCAGGACCACATCCAGAGCCGGATCGTCTAC    420
M                   GTGGGCCGGCGGTTCCTGGTGAACAGGGTTCAGGACCACATCCAGAGCCGGATCGTCTAC    420
                    ************************************************************

XM_064479318.1      TACCTAATGAACATCCACGTGCAG    444
H                   TACCTAATGAACATCCACGTGCAG    444
M                   TACCTAATGAACATCCACGTGCAG    444
                    ************************
```

Figure S8: Comparative alignment of coding DNA sequence (CDS) fragments of the *PFKFB3* gene (444-bp) between healthy and metritis she-camel relative to the corresponding reference sequence retrieved from GenBank (XM_064479318.1). H= healthy; and M= Metritis. Asterisks represent similarity.

```
XM_010983554.3      CTGAGCAGCGAGGTGTTGGTGGGGGGCTTGATGTCCCCCTTCGACCAGTCGGGTTTGGGG    60
H                   CTGAGCAGCGAGGTGTTGGTGGGGGGCTTGATGTCCCCCTTCGACCAGTCGGGTTTGGGG    60
M                   CTGAGCAGCGAGGTGTTGGTGGGGGGCTTGATGTCCCCCTTCGACCAGTCGGGTTTGGGG    60
                    ************************************************************

XM_010983554.3      GCTGAAGAGAGCCTAGGTCTCTTAGACGACTACCTGGAGGTGGCCAAGCACTTCAAACCT    120
H                   GCTGAAGAGAGCCTAGGTCTCTTAGACGACTACCTGGAGGTGGCCAAGCACTTCAAACCT    120
M                   GCTGAAGAGAGCCTAGGTCTCTTAGACGACTACCTGGAGGTGGCCAAGCACTTCAAACCT    120
                    ************************************************************

XM_010983554.3      CATGGGTTCTCCAGCGACAAAGCTAAGGCGGGCTCCTCCGAATGGCTGGCTGTGGATGGG    180
H                   CATGGGTTCTCCAGCGACAAAGCTAAGGCGGGCTCCTCCGAATGGCTGGCTGTGGATGGG    180
M                   CATGGGTTCTCCAGCGACAAAGCTAAGGCGGGCTCCTCCGAATGGCTGGCTGTGGATGGG    180
                    ************************************************************

XM_010983554.3      TTGGTCAGTGCCTCAGACAACGGCAAGGAGGATGCTTTCTCCGGGACAGATTGGATGGTG    240
H                   TTGGTCAGTGCCTCAGACAACGGCAAGGAGGATGCTTTCTCCGGGACAGATTGGATGGTG    240
M                   TTGGTCAGTGCCTCAGACAACGGAAAGGAGGATGCTTTCTCCGGGACAGATTGGATGGTG    240
                    *********************** ************************************

XM_010983554.3      GAGAAAATGGACCTGAAGGAGTTCGACTTTGATGCCCTGTTGGGTATAGATGACCTGGAA    300
H                   GAGAAAATGGACCTGAAGGAGTTCGACTTTGATGCCCTGTTGGGTATAGATGACCTGGAA    300
M                   GAGAAAATGGACCTGAAGGAGTTCGACTTTGATGCCCTGTTGGGTATAGATGACCTGGAA    300
                    ************************************************************

XM_010983554.3      ACCATGCCAGATGAGCTTTTGGCCACGTTGGATGACACATGTGATCTCTTTGACCCCCTA    360
H                   ACCATGCCAGATGAGCTTTTGGCCACGTTGGATGACACATGTGATCTCTTTGACCCCCTA    360
M                   ACCATGCCAGATGAGCTTTTGGCCACGTTGGATGACACATGTGATCTCTTTGACCCCCTA    360
                    ************************************************************

XM_010983554.3      TTCCAGGAAACTAATAAGGAGCCCCCCCAGATAGTGAACCCAATTGGCCATCTCCCAGA     419
H                   TTCCAGGAAACTAATAAGGAGCCCCCCCAGATAGTGAACCCAATTGGCCATCTCCCAGA     419
M                   TTCCAGGAAACTAATAAGGAGCCCCCCCAGATAGTGAACCCAATTGGCCATCTCCCAGA     419
                    ***********************************************************
```

Figure S9: Comparative alignment of coding DNA sequence (CDS) fragments of the *ATF4* gene (419-bp) between healthy and metritis she-camel relative to the corresponding reference sequence retrieved from GenBank (XM_010983554.3). H= healthy; and M= Metritis. Asterisks represent similarity.

```
XM_010997221.3      GTGGCCGCGCAGCGCCAGGACCGCCTCACCGTGGGGGTGTACGAGTCGGCCAAGCTGATG   60
H                   GTGGCCGCGCAGCGCCAGGACCGCCTCACCGTGGGGGTGTACGAGTCGGCCAAGCTGATG   60
M                   GTGGCCGCGCAGCGCCAGGACCGCCTCACCGTGGGGGTGTACGAGTCGGCCAAGCTGATG   60
                    ************************************************************

XM_010997221.3      AATGTGGACCCTGACAGCGTGGTCCTGTGTCTCCTGGCCATTGATGAGGAGGAAGAAGAT   120
H                   AATGTGGACCCTGACAGCGTGGTCCTGTGTCTCCTGGCCATTGATGAGGAGGAAGAAGAT   120
M                   AATGTGGACCCTGACAGCGTGGTCCTGTGCCTCCTGGCCATTGATGAGGAGGAAGAAGAT   120
                    ***************************** ******************************

XM_010997221.3      GACATCGCCCTGCAGATCCACTTCACGCTCATCCAGTCTTTCTGCTGTGACAACGACATC   180
H                   GACATCGCCCTGCAGATCCACTTCACGCTCATCCAGTCTTTCTGCTGTGACAACGACATC   180
M                   GACATCGCCCTGCAGATCCACTTCACGCTCATCCAGTCTTTCTGCTGTGACAACGACATC   180
                    ************************************************************

XM_010997221.3      GACATCGTGCGGGTGTCGGGCATGCAGCGGCTGGCACAGCTGCTGGGCGAGCCAGCCGAG   240
H                   GACATCGTGCGGGTGTCGGGCATGCAGCGGCTGGCACAGCTGCTGGGCGAGCCAGCCGAG   240
M                   GACATCGTGCGGGTGTCGGGCATGCAGCGGCTGGCACAGCTGCTGGGCGAGCCAGCCGAG   240
                    ************************************************************

XM_010997221.3      ACCCAGGGCACCACCGAGGCTCGGGACCTGCACTGCCTCCTGGTCACGAACCCTCACACA   300
H                   ACCCAGGGCACCACCGAGGCTCGGGACCTGCACTGCCTCCTGGTCACGAACCCTCACACA   300
M                   ACCCAGGGCACCACCGAGGCTCGGGACCTGCACTGCCTCCTGGTCACGAACCCTCACACA   300
                    ************************************************************

XM_010997221.3      GATGCCTGGAAAAGCCACGGTCTGGTGGAGGTGGCCAGTTACTGTGAAGAGAGCCGGGAG   360
H                   GATGCCTGGAAAAGCCACGGTCTGGTGGAGGTGGCCAGTTACTGTGAAGAGAGCCGGGAG   360
M                   GATGCCTGGAAAAGCCACGGTCTGGTGGAGGTGGCCAGTTACTGTGAAGAGAGCCGGGAG   360
                    ************************************************************

XM_010997221.3      AACAACCAGTGGGTC   375
H                   AACAACCAGTGGGTC   375
M                   AACAACCAGTGGGTC   375
                    ***************
```

Figure S10: Comparative alignment of coding DNA sequence (CDS) fragments of the *GADD45B* gene (375-bp) between healthy and metritis she-camel relative to the corresponding reference sequence retrieved from GenBank (XM_010997221.3). H= healthy; and M= Metritis. Asterisks represent similarity.

```
XM_031465945.2      GTCAGAAAGCCCGTTGTGTCCACCATCTCCAATGGAGGTTACCTGCAGGGGAATGTTCAA    60
H                   GTCAGAAAGCCCGTTGTGTCCACCATCTCCAGTGGAGGTTACCTGCAGGGGAATGTTCAA    60
M                   GTCAGAAAGCCCGTTGTGTCCACCATCTCCAATGGAGGTTACCTGCAGGGGAATGTTCAA    60
                    ******************************* ****************************

XM_031465945.2      GGGAGGCTGCCTTCCCTGGGCAGCAAGGAGCCACCTGGGCAGGAGAGAGTTGTGCTCAAG    120
H                   GGGAGGCTGCCTTCCCTGGGCAGCAAGGAGCCACCTGGGCAGGAGAGAGTTGTGCTCAAG    120
M                   GGGAGGCTGCCTTCCCTGGGCAGCAAGGAGCCACCTGGGCAGGAGAGAGTTGTGCTCAAG    120
                    ************************************************************

XM_031465945.2      AAGAAAATCACTCTGTTGAGAGGAATCTCCATCATCATCGGCACCATCATTGGAGCAGGA    180
H                   AAGAAAATCACTCTGTTGAGAGGAATCTCCATCATCATCGGCACCATCATTGGAGCAGGA    180
M                   AAGAAAATCACTCTGTTGAGAGGAATCTCCATCATCATCGGCACCATCATTGGAGCAGGA    180
                    ************************************************************

XM_031465945.2      ATCTTCATCTCTCCCAAGGGCATCCTCCAGAACACGGGCAGCGTGGGCTTGTCTCTGGTT    240
H                   ATCTTCATCTCTCCCAAGGGCATCCTCCAGAACACGGGCAGCGTGGGCTTGTCTCTGGTT    240
M                   ATCTTCATCTCTCCCAAGGGCATCCTCCAGAACACGGGCAGCGTGGGCTTGTCTCTGGTT    240
                    ************************************************************

XM_031465945.2      ATCTGGACGGTCTGTGGGGTCCTGTCACTGTTTGGAGCCTTGTCCTATGCTGAATTGGGA    300
H                   ATCTGGACGGTCTGTGGGGTCCTGTCACTGTTTGGAGCCTTGTCCTATGCTGAATTGGGA    300
M                   ATCTGGACGGTCTGTGGGGTCCTGTCACTGTTTGGAGCCTTGTCCTATGCTGAATTGGGA    300
                    ************************************************************

XM_031465945.2      ACCAGTATAAAGAAATCTGGCGGTCATTACACATACATCCTGGAAGTCTTTGGCCCATTA    360
H                   ACCAGTATAAAGAAATCTGGCGGTCATTACACATACATCCTGGAAGTCTTTGGCCCATTA    360
M                   ACCAGTATAAAGAAATCTGGCGGTCATTACACATACATCCTGGAAGTCTTTGGCCCATTA    360
                    ************************************************************

XM_031465945.2      CCAGCTTTTGTGCGAGTCTGGGTGGAACTGCTCGTAATACGCCCTGCAGCCACTGCTGTG    420
H                   CCAGCTTTTGTGCGAGTCTGGGTGGAACTGCTCATAATACGCCCTGCAGCCACTGCTGTG    420
M                   CCAGCTTTTGTGCGAGTCTGGGTGGAACTGCTCGTAATACGCCCTGCAGCCACTGCTGTG    420
                    ********************************* **************************

XM_031465945.2      ATATCTCTGGCATTTGGACGCTACA 445
H                   ATATCTCTGGCATTTGGACGCTACA 445
M                   ATATCTCTGGCATTTGGACGCTACA 445
                    *************************
```

Figure S11: Comparative alignment of coding DNA sequence (CDS) fragments of the *SLC7A11* gene (445-bp) between healthy and metritis she-camel relative to the corresponding reference sequence retrieved from GenBank (XM_031465945.2). H= healthy; and M= Metritis. Asterisks represent similarity.

```
XM_010997880.3      CCTTATGCCTCTCGAAGAATGTGGTTGACAAGTGCATTGTGGTCCAGTTCCAGTCAAGCA    60
H                   CCTTATGCCTCTCGAAGAATGTGGTTGACAAGTGCATTGTGGTCCAGTTCCAGTCAAGCA    60
M                   CCTTATGCCTCTCGAAGAATGTGGTTGACAAGTGCATTGTGGTCCAGTTCCAGTCAAGCA    60
                    ************************************************************

XM_010997880.3      AAATTCACCTTTAGCACCAGTTCCTCATACAATTTCCCTGCTGTCACCCAGCATGCGCCC    120
H                   AAATTCACCTTTAGCACCAGTTCCTCATACAATTTCCCTGCTGTCACCCAGCATGCGCCC    120
M                   AAATTCACCTTTAGCACCAGTTCCTCATACAATTTCCCTGCTGTCACCCAGCATGCGCCC    120
                    ************************************************************

XM_010997880.3      TATTTTAAGGGTACAGCCGTTGTCAATGGAGAGTTCAAAGAACTAAGCCTTGATGACTTT    180
H                   TATTTTAAGGGTACAGCCGTTGTCAATGGAGAGTTCAAAGAACTAAGCCTTGAAGACTTT    180
M                   TATTTTAAGGGTACAGCCGTTGTCAATGGAGAGTTCAAAGAACTAAGCCTTGATGACTTT    180
                    ***************************************************** ******

XM_010997880.3      AAGGGGAAATATTTGGTGCTTTTCTTCTATCCTTTGGATTTCACCTTTGTGTGTCCTACA    240
H                   AAGGGGAAATATTTGGTGCTTTTCTTCTATCCTTTGGATTTCACCTTTGTGTGTCCTACA    240
M                   AAGGGGAAATATTTGGTGCTTTTCTTCTATCCTTTGGATTTCACCTTTGTGTGTCCTACA    240
                    ************************************************************

XM_010997880.3      GAAATTATAGCTTTCAGTGACAAAGCCAATGAATTTCATGATGTGAACTGTGAAGTCATT    300
H                   GAAATTATAGCTTTCAGTGACAAAGCCAATGAATTTCATGATGTGAACTGTGAAGTCATT    300
M                   GAAATTATAGCTTTCAGTGACAAAGCCAATGAATTTCATGATGTGAACTGTGAAGTCATT    300
                    ************************************************************

XM_010997880.3      GCAGTGTCAGTGGACTCCCACTTCAGCCATCTCGCCTGGATAAACACACCAAGGAAGAAT    360
H                   GCAGTGTCAGTGGACTCCCACTTCAGCCATCTCGCCTGGATAAACACACCAAGGAAGAAT    360
M                   GCAGTGTCAGTGGACTCCCACTTCAGCCATCTCGCCTGGATAAACACACCAAGGAAGAAT    360
                    ************************************************************

XM_010997880.3      GGCGGTTTGGGCCACATGAACATCGCACTGTTGTCAGATTTGACTAAACAGATTTCCCGA    420
H                   GGCGGTTTGGGCCACATGAACATCGCACTGTTGTCAGATTTGACTAAACAGATTTCCCGA    420
M                   GGCGGTTTGGGCCACATGAACATCGCACTGTTGTCAGATTTGACTAAACAGATTTCCCGA    420
                    ************************************************************

XM_010997880.3      GACTACGGTGTGCTGTTGGAAGGTGCTGGTCTGGCCCTA     459
H                   GACTACGGTGTGCTGTTGGAAGGTGCTGGTCTGGCCCTA     459
M                   GACTACGGTGTGCTGTTGGAAGGTGCTGGTCTGGCCCTA     459
                    ***************************************
```

Figure S12: Comparative alignment of coding DNA sequence (CDS) fragments of the *PRDX3* gene (459-bp) between healthy and metritis she-camel relative to the corresponding reference sequence retrieved from GenBank (XM_010997880.3). H= healthy; and M= Metritis. Asterisks represent similarity.

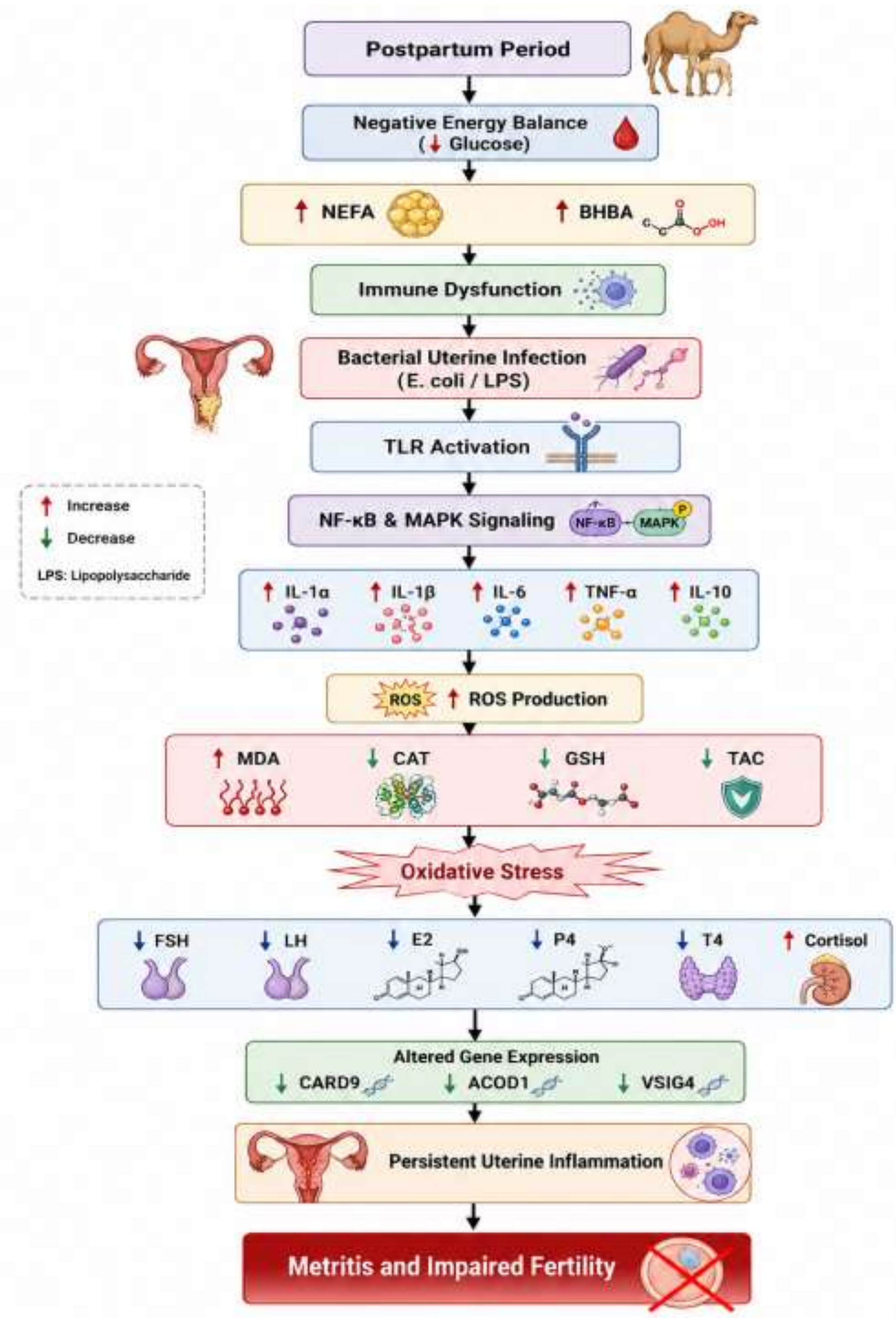

Figure S13: Proposed mechanism linking metabolic imbalance, inflammation, oxidative stress, hormonal disturbances and gene expression to metritis in postpartum camels.
